# Synthesis and Structure Determination of Substituted Thiazole Derivatives as EGFR/BRAF^V600E^ Dual Inhibitors Endowed with Antiproliferative Activity

**DOI:** 10.3390/ph16071014

**Published:** 2023-07-17

**Authors:** Lamya H. Al-Wahaibi, Essmat M. El-Sheref, Alaa A. Hassan, S. Bräse, M. Nieger, Bahaa G. M. Youssif, Mahmoud A. A. Ibrahim, Hendawy N. Tawfeek

**Affiliations:** 1 Department of Chemistry, College of Sciences, Princess Nourah Bint Abdulrahman University, Riyadh 11564, Saudi Arabia; 2 Chemistry Department, Faculty of Science, Minia University, El Minia 61519, Egypt; essmat_elsheref@mu.edu.eg (E.M.E.-S.); alaahassan2001@mu.edu.eg (A.A.H.); m.ibrahim@compchem.net (M.A.A.I.); hendawy1976@yahoo.com (H.N.T.); 3 Institute of Biological and Chemical Systems, IBCS-FMS, Karlsruhe Institute of Technology, 76131 Karlsruhe, Germany; 4 Department of Chemistry, University of Helsinki, P.O. Box 55 (A. I. Virtasen aukio 1), 00014 Helsinki, Finland; martin.nieger@helsinki.fi; 5 Pharmaceutical Organic Chemistry Department, Faculty of Pharmacy, Assiut University, Assiut 71526, Egypt; 6 School of Health Sciences, University of KwaZulu-Natal, Westville Campus, Durban 4000, South Africa; 7 Unit of Occupational of Safety and Health, Administration Office of Minia University, El-Minia 61519, Egypt

**Keywords:** thiazole, thiosemicarbazide, X-ray, viability, antiproliferative, molecular modeling

## Abstract

2,3,4-trisubstituted thiazoles **3a**–**i**, having a methyl group in position four, were synthesized by the reaction of 1,4-disubstituted thiosemicarbazides with chloroacetone in ethyl acetate/Et_3_N at room temperature or in ethanol under reflux. The structures of new compounds were determined using NMR spectroscopy, mass spectrometry, and elemental analyses. Moreover, the structure of compound **3a** was unambiguously confirmed with X-ray analysis. The cell viability assay of **3a**–**i** at 50 µM was greater than 87%, and none of the tested substances were cytotoxic. Compounds **3a**–**i** demonstrated good antiproliferative activity, with GI_50_ values ranging from 37 to 86 nM against the four tested human cancer cell lines, compared to the reference erlotinib, which had a GI_50_ value of 33 nM. The most potent derivatives were found to be compounds **3a**, **3c**, **3d**, and **3f**, with GI_50_ values ranging from 37 nM to 54 nM. The EGFR-TK and BRAF^V600E^ inhibitory assays’ results matched the antiproliferative assay’s results, with the most potent derivatives, as antiproliferative agents, also being the most potent EGFR and BRAF^V600E^ inhibitors. The docking computations were employed to investigate the docking modes and scores of compounds **3a**, **3c**, **3d**, and **3f** toward BRAF^V600E^ and EGFR. Docking computations demonstrated the good affinity of compound **3f** against BRAF^V600E^ and EGFR, with values of −8.7 and −8.5 kcal/mol, respectively.

## 1. Introduction

Kinases control many essential cancer processes, including tumor growth, metastasis, neovascularization, and treatment resistance. Hence, the development of kinase inhibitors has become a top priority, with several of them receiving FDA approval for a variety of cancer purposes [1,2,3,4,5].

One approach for simultaneously inhibiting two or more targets is combination chemotherapy. However, two or more drugs’ pharmacokinetic profiles and metabolic stabilities frequently differ. Furthermore, drug–drug interactions may occur during combination chemotherapy [6,7,8]. An alternative method for addressing these issues is to use a single drug to suppress two or more targets [9,10,11,12]. This approach may even make patients’ treatment easier. The FDA has approved many dual-target or multi-target cancer treatments. Dasatinib is a multi-targeted kinase inhibitor that can potentially be a highly effective anticancer medication [13,14,15,16,17].

The acquired BRAF^V600E^ mutation was suggested as a resistance mechanism after therapy with an EGFR inhibitor [18,19]. The development of resistance in colorectal cancer was also linked to the feedback stimulation of EGFR signaling [20,21,22]. Additionally, BRAF inhibition can cause EGFR to become active, promoting tumor growth [23,24]. A BRAF/EGFR combination was used to adopt these issues. In a number of studies, the BRAF/EGFR combination was found to have a significant therapeutic effect in patients with metastatic colorectal cancer that had BRAF^V600E^ mutations [18,25,26]. As a result, sequentially inhibiting the two kinases may provide a solution to the EGFR activation problem.

**Figure 1:**
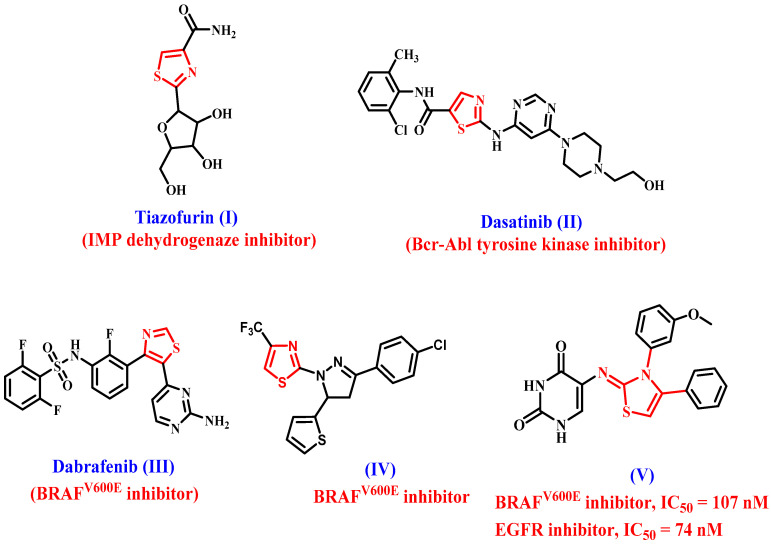
Structures of thiazole-based anticancer drugs **I**–**III** and compounds **IV** and **V**.

Thiazole and its derivatives are also among the most active chemicals, ranking first in anticancer activity [27,28,29]. As well, thiazole-containing molecules were identified in a number of therapeutically available anticancer medicines (Figure 1), including tiazofurin (**I**) [30], dasatinib (**II**) [31,32], and dabrafenib (**III**) [33,34].

Abdel-Maksoud et al. [35] investigated several thiazole-based compounds as potential BRAF^V600E^ inhibitors. Compound **IV** (Figure 1) had the most potent antiproliferative activity, with a competitive BRAF^V600E^ inhibitory action (IC_50_ = 0.05 µM). Furthermore, compound **IV** significantly affected dose-dependent apoptosis.

We recently reported on the design and synthesis of two series of thiazole-based compounds as potent antiproliferative agents targeting EGFR and BRAF^V600E^ [36]. Compound **V** (Figure 1) was shown to be the most potent derivative of all synthesized compounds, with a GI_50_ value of 0.90 µM against the four evaluated cancer cell lines when compared to the reference doxorubicin (GI_50_ = 1.10 µM). Compound **V** inhibited EGFR and BRAF^V600E^ with IC_50_ values of 74 ± 7 and 107 ± 10 nM, respectively, and was more effective than erlotinib against EGFR (IC_50_ = 80 nM).

Moreover, the sulfonamide moiety is commonly employed in medicinal chemistry as efficient bioisosteres of the carboxylic group [37,38]. The sulfonamide motif could build a network of hydrogen bonds similar to the carboxylic group. As the carboxylic group’s bioisosteres, it could avoid some of the carboxylic group’s limitations, such as metabolic instability, toxicity, and limited passive diffusion across biological membranes [37]. As a result, the sulfonamide moiety gained popularity in medicinal chemistry, and a wide range of sulfonamide derivatives with diverse biological properties, such as anticancer activity [39,40,41], were produced.

**Figure 2:**
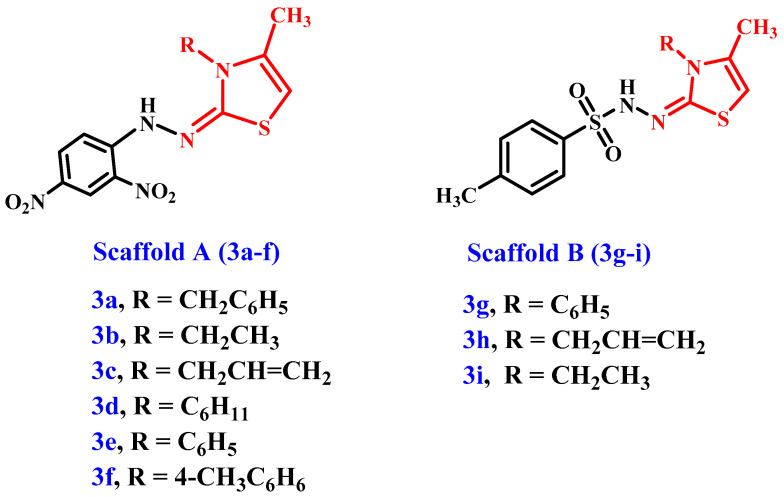
The new target compounds’ **3a**–**i** structures.

In light of the aforementioned information, and as part of our enduring effort to develop potent antiproliferative agents that are dual inhibitors of EGFR and BRAF^V600E^ [42,43,44,45], we describe the synthesis of a new set of thiazole-based compounds **3a**–**i** (Figure 2) in this article as antiproliferative agents that target EGFR and/or mutant BRAF. Scaffold A and B molecules had a methyl group in position 4, a hydrazo group in position 2, a physiologically active tosyl group for the scaffold B compounds, and a 2,4-dinitrophenyl group for the scaffold A compounds (Figure 2). The cell viability of the novel derivatives was tested against a normal human mammary gland epithelial (MCF-10A) cell line. The antiproliferative action of **3a**–**i** was tested on a panel of four human cancer cell lines. The ability to inhibit EGFR and mutant BRAF was further assessed for the most active antiproliferative derivatives. Finally, the most potent compounds’ binding modes and docking scores toward BRAF^V600E^ and EGFR targets were investigated.

## 2. Results and Discussion

### 2.1. Chemistry

This study aimed to develop new thiazole derivatives in a straightforward manner. Indeed, a novel series of *(Z)*-3-substituted-2-(2-substituted)-hydrazinylidene)-4-methyl-2,3-dihydrothiazoles **3a**–**i** were synthesized in an excellent yield of 78–99% via a mixture of substituted hydrazine-carbothioamides **1a**–**i** [46,47,48,49] and chloroacetone (**2**) in ethyl acetate as a solvent. Et_3_N catalyzed the reaction by stirring overnight at room temperature or refluxing in ethanol for 6–10 hrs (Scheme 1). For example, compound **3a** was obtained in (AcOEt/Et_3_N: 98%) and (EtOH: 85%) yield after recrystallization (Table 1). The structure assignment for all obtained products **3a**–**i** was confirmed by IR, NMR analysis (Supplementary File Figures S2–S32) of the expected chemical shifts, mass spectrometry, elemental analysis, and X-ray crystallography.

**Figure 3:**
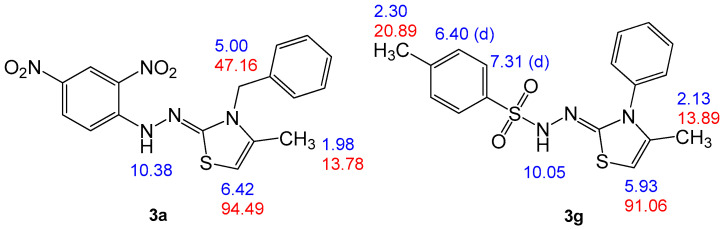
Structure elucidation of compounds **3a** and **3g**.

Compound **3a** was chosen as a representative example, which was assigned as (*Z*)-3-benzyl-2-(2-(2,4-dinitrophenyl)hydrazinylidene)-4-methyl-2,3-dihydrothiazole and exhibited a molecular formula C_17_H_15_N_5_O_4_S with mass *m/z* (385). Elemental analysis and mass spectrometry confirmed that **3a** was formed by an interaction between one mole of *N*-benzyl-2-(2,4-dinitrophenyl)hydrazinecarbothioamide (**1a**) and one mole of chloroacetone (**2**), with the loss of a molecule of HCl and another molecule of H_2_O. FTIR analysis of thiazole compound **3a** showed different peaks at 3286 cm^−1^ due to hydrazo-NH stretching, 3085 cm^−1^ for aromatic stretching-CH, and 2978 cm^−1^ for aliphatic-CH as well as two peaks at 1609 and 1548 cm^−1^ for C=N and C=C_,_ respectively. IR showed a peak at 1399 and 1117 cm^−1^ for the NO_2_ group. Further, in the ^1^H NMR spectrum of **3a**, five singlet signals were distinguished at δ_H_ = 1.98, 5, 6.42, 8.68, and 10.38 ppm, which were assigned as CH_3_, benzyl-CH_2_, thiazole-H, 2,4-dinitrophenyl-H-3, and hydrazono-NH, respectively (Figure 3). The ^13^C NMR spectrum for compound **3a** revealed signals at δ_C_ = 13.78, 47.16, and 94.49 ppm, which were assigned as CH_3_, benzyl-CH_2_, and thiazole-C5, respectively. Moreover, C4 and C2 gave signals at δ_C_ = 135.13 and 164.14 ppm, respectively.

To make comparable results, we chose another compound, **3g**, which was assigned as (*Z*)-4-methyl-*N*′-(4-methyl-3-phenylthiazol-2(3*H*)-ylidene) benzenesulfonohydrazide, with a molecular formula C_17_H_17_N_3_O_2_S_2_ (*m/z* = 359). Compound **3g** is analogous for the above compound by replacing the benzyl group and 2,4-dinitrophenyl with phenyl and 4-methyl-benzensulfonoyl groups, respectively. The ^1^H NMR for this compound is similar for compound **3a** unless the 1,4-disubstituted benzene gives characteristic signals as a doublet at δ_H_ = 6.40 (d, 2H) and 7.31 ppm (d, 2H), which were assigned as H-*m* and H-*o*, respectively. Moreover, by comparing the data for the two compounds, as shown in Figure 3, it is clear that the reaction behaves the same with the difference in the substitutes and that the difference is a slight difference in the chemical shift’s results for the difference only in the nature of the substituted groups.

Another example is compound **3b,** which was assigned as (*Z*)-2-(2-(2,4-dinitrophenyl)-hydrazinylidene)-3-ethyl-4-methyl-2,3-dihydrothiazole and has the same spectral data as compound **3a** except that the benzyl group was replaced with ethyl, which gives two characteristic signals as triplet–quartet and appears in its ^1^H NMR spectrum at δ_H_ = 1.28–1.38 (t, *J* = 3 Hz; 3H, ethyl-CH_3_) and 3.88–3.98 ppm (q, *J* = 3 Hz; 2H, ethyl-CH_3_) and was confirmed from its ^13^C NMR spectrum, with two signals at δ_H_ = 12.98 (ethyl-CH_3_) and 30.67 ppm (ethyl-CH_2_).

**Figure 4:**
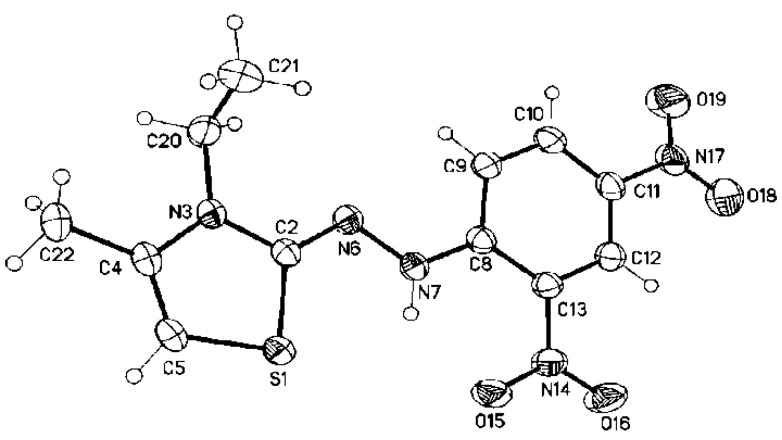
The crystal structure of *(Z)*-2-(2-(2,4-dinitrophenyl)hydrazinylidene)-3-ethyl-4-methyl-2,3-dihydrothiazole **3b**.

Furthermore, the structures for the obtained products were confirmed via X-ray crystallography. Moreover, the X-ray measurements of compound **3b** showed that the molecule (except the C-atom of the ethyl substituent, C21) is virtual planar. The aromatic ring is coplanar with the thiazole ring, and the ethyl group has the hours conformation structure. The angle between the thiazole and the aromatic ring is 6.37(7)°, between the thiazole and the hydrazinylidene moiety is 2.98(14)°, and between the aromatic ring and the hydrazinylidene moiety is 6.56(9)° (angle between the L.S. planes of the moieties). In addition, the geometric structure around the exocyclic C=N has cissoid geometry concerning the thiazole *S*-atom and the hydrazo-group (Figure 4). The geometrical parameters (selected bond distance, bond angles, and dihedral angles; see Table 2) are in good correlation with the theoretical values.

**Scheme 2:**
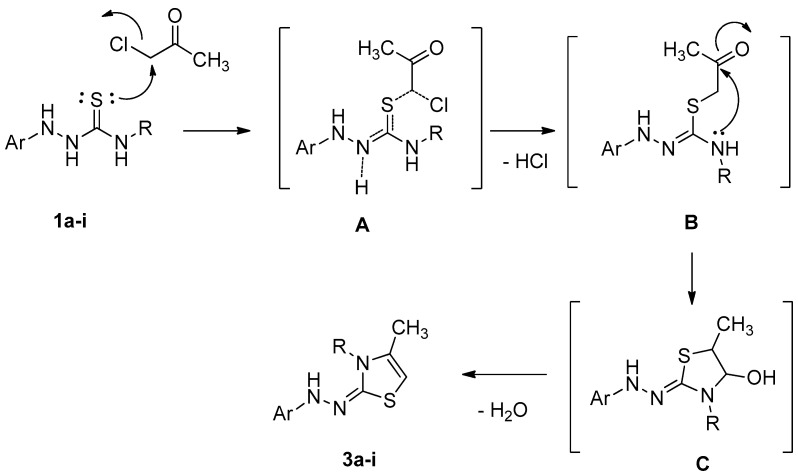
The hypothesized mechanism for the synthesis of thiazole compounds **3a–i**.

Based on the above results and the X-ray confirmation of our obtained products, the proposed mechanism is as follows. First, the nucleophilic attack of sulfur on the primary carbon atom results in the formation of the intermediate, **B** (S-alkylation), via the transition state, **A**. Another nucleophilic attack on the nitrogen atom on the carbonyl carbon gives the intermediate, **C**, followed by water molecule (dehydration) loss to give the target product. The reaction mechanism proceeds via the S_N_^2^ reaction type (Scheme 2).

### 2.2. Biology

#### 2.2.1. Cell Viability Assay

The human mammary gland epithelial (MCF-10A) cell line was used to test the viability of the novel compounds [50,51]. After four days of incubation on MCF-10A cells, the vitality of compounds **3a**–**i** was determined using the MTT method. According to Table 3, the cell viability at 50 µM was greater than 87% for all tested agents, and none of the tested substances were cytotoxic.

#### 2.2.2. Antiproliferative Assay

The MTT assay was used to investigate the antiproliferative activity of **3a**–**i** against four human cancer cell lines: the colon cancer (HT-29) cell line, pancreatic cancer (Panc-1) cell line, lung cancer (A-549) cell line, and breast cancer (MCF-7) cell line, using erlotinib as the reference [52,53]. Table 3 shows the median inhibitory concentration (IC_50_).

In general, the examined compounds **3a**–**i** displayed good antiproliferative activity, with average IC_50_ (GI_50_) values ranging from 37 to 86 nM against the four tested human cancer cell lines, compared to the reference erlotinib (GI_50_ = 33 nM).

The most potent derivatives were compounds **3a**, **3c**, **3d**, and **3f**, with GI_50_ values ranging from 37 nM to 54 nM. Compound **3f** (Ar = 2,4-di-NO_2_-C_6_H_3_, R = 4-CH_3_-C_6_H_5_) was the most potent derivative of all synthesized compounds, with a GI_50_ value of 37 nM against the four tested human cancer cell lines, comparable to the reference erlotinib (GI_50_ = 33 nM). By replacing the *p*-tolyl group in compound **3f** with a cyclohexyl moiety, compound **3d** (Ar = 2,4-di-NO_2_-C_6_H_3_, R = C_6_H_11_) was found to be the second-most potent compound, with a GI_50_ value of 46 nM, being 1.3-fold less potent than compound **3f**, demonstrating the importance of the *p*-tolyl moiety in antiproliferative activity.

The benzyl derivative, **3a** (Ar = 2,4-di-NO_2_-C_6_H_3_, R = CH_2_-C_6_H_5_), was less potent than **3f** and **3d**, with a GI_50_ value of 48 nM against the tested four cancer cell lines, while the allyl derivatives, **3c** (Ar = 2,4-di-NO_2_-C_6_H_3_, R = CH_2_CH = CH_2_), showed moderate antiproliferative activity, with a GI_50_ value more than 50 nM. These findings show that allyl and benzyl groups are not preferred for the antiproliferative activity of scaffold A compounds **3a**–**f**.

The remaining scaffold A compounds, **3b** (Ar = 2,4-di-NO_2_-C_6_H_3_, R = CH_2_CH_3_) and **3e** (Ar = 2,4-di-NO_2_-C_6_H_3_, R = C_6_H_5_), had GI_50_ values of 80 nM and 72 nM, respectively. Compounds **3b** and **3e** were 2.2- and 2-fold less potent than **3f**, respectively, indicating weak antiproliferative activity (Table 3).

With GI_50_ values of 60 nM, 65 nM, and 86 nM, scaffold B compounds **3g**, **3h**, and **3i** demonstrated moderate-to-weak antiproliferative activity. Compound **3i** (Ar = *p*-CH_3_-C_6_H_4_-SO_2_, R = CH_2_CH_3_) was the least potent derivative of any of the synthesized compounds, with a GI_50_ value of 86 nM, which is less potent than its congeners, **3b** (scaffold A), which has the same structure, but the aryl moiety was *p*-CH_3_-C_6_H_4_-SO_2_, while in **3b** it was 2,4-di-NO_2_-C_6_H_3_. These findings demonstrated that 2,4-di-NO_2_-C_6_H_3_ significantly affects the antiproliferative action of the newly synthesized compounds.

#### 2.2.3. Assay for EGFR Inhibition

The most promising antiproliferative compounds, **3a**, **3c**, **3d**, and **3f**, were further evaluated for their suppressive impact on EGFR as a probable target for their mechanism of action [50,54,55]. Table 4 compares the IC_50_ values to erlotinib, which worked as a control.

The EGFR-TK inhibitory assay results matched the antiproliferative assay results, with the most potent derivatives, as antiproliferative agents, also being the most potent EGFR inhibitors. Compounds **3a**, **3c**, **3d**, and **3f** inhibited EGFR, with IC_50_ values ranging from 89 to 98 nM, but the tested compounds were less potent than erlotinib (IC_50_ = 80 nM). The most potent antiproliferative agent, compound **3f** (Ar = 2,4-di-NO_2_-C_6_H_3_, R = 4-CH_3_-C_6_H_5_), was also the most potent EGFR inhibitor, with an IC_50_ value of 89 ± 7, being 1.1-fold less potent than standard erlotinib.

Compounds **3a** (Ar = 2,4-di-NO_2_-C_6_H_3_, R = CH_2_-C_6_H_5_) and **3d** (Ar = 2,4-di-NO_2_-C_6_H_3_, R = C_6_H_11_) ranked third and second in EGFR suppression, with IC_50_ values of 93 ± 8 and 91 ± 7 nM, respectively, being 1.15-fold less potent than erlotinib (IC_50_ = 80 ± 5 nM). These findings suggest that EGFR-TK could be a molecular target for the tested compound’s antiproliferative action.

#### 2.2.4. BRAF^V600E^ Inhibitory Assay

Derivatives **3a**, **3c**, **3d**, and **3f** were further investigated as possible BRAF^V600E^ inhibitors [56]. Table 4 displays the IC_50_ values compared to erlotinib, which was used as a control. According to Table 4, the evaluated derivatives had a promising BRAF^V600E^ suppressive action, with IC_50_ values ranging from 93 to 126 nM, making them approximately 1.5-fold less effective than erlotinib (IC_50_ = 60 nM). Compound **3f**, the most potent derivative in the antiproliferative and EGFR suppressive assays, was also the most effective derivative as anti-BRAF^V600E^ (IC_50_ = 93 ± 8 nM). These findings show that compound **3a** has potent antiproliferative activity as a dual EGFR/BRAF^V600E^ inhibitor, implying that further structural modifications may be required to obtain a more potent lead compound for future development.

### 2.3. In Silico Study

AutoDock4.2.6 software was used to investigate the binding scores and poses of compounds **3a**, **3c**, **3d**, and **3f** against BRAF^V600E^ and EGFR. The estimated docking features and scores are listed in Table 5. As tabulated in Table 5, all inspected compounds revealed good docking scores against BRAF^V600E^ and EGFR targets**,** ranging from −7.8 to −8.7 kcal/mol and from −7.9 to −8.5 kcal/mol, respectively. The good docking scores of the inspected compounds toward BRAF^V600E^ and EGFR may be imputed to their capability of forming H-bonds and vdW, pi-based, and hydrophobic interactions with the proximal residues within the active sites of the investigated targets (Table 5).

**Figure 5:**
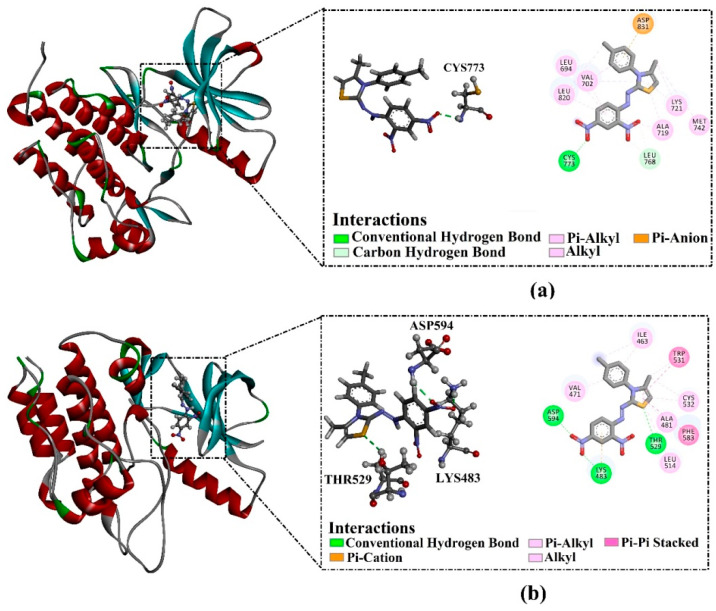
Molecular interactions of compound **3f** within (**a**) EGFR and (**b**) BRAF^V600E^ active sites.

Compound **3f** demonstrated superior docking scores of −8.7 and −8.5 kcal/mol against BRAF^V600E^ and EGFR, respectively. Inspecting the docking mode of compound **3f** with the EGFR active site unveiled that this compound formed one H-bond with CYS773 (2.18 Å). Moreover, compound **3f** exhibited two carbon–hydrogen bonds with LEU768 and pi–anion interaction with ASP831. On the other hand, compound **3f**, complexed with BRAF^V600E^, demonstrated three H-bonds with LYS483 (2.15 Å), THR529 (2.38 Å), and ASP594 (2.12 Å). Additionally, compound **3f** established pi–cation interaction with LYS483 and pi–pi stacking interaction with PHE583 and TRP531 residues (Figure 5).

Compound **3d** showed the second-lowest docking score, with values of −8.5 and −8.1 kcal/mol against BRAF^V600E^ and EGFR, respectively. Compound **3d** displayed one hydrogen bond with the CYS773 (2.17 Å) within the active site of EGFR. However, compound **3d** demonstrated four H-bonds with the LYS483 (1.93Å), GLY596 (2.32 Å), THR529 (2.66 Å), and ASP594 (2.14 Å) of BRAF^V600E^.

Compound **3a** exposed the third-lowest docking score, with values of −8.4 and −8.0 kcal/mol against BRAF^V600E^ and EGFR, respectively (Table 5). Compound **3a** made one H-bond with **the** CYS773 (2.21 Å) of EGFR, while compound **3a** exhibited three H-bonds with the LYS483 (2.15 Å), THR529 (2.38 Å), and ASP594 (2.12 Å) within the BRAF^V600E^ binding pocket.

Compound **3c** also unveiled good docking scores, with values of −7.8 and −7.9 kcal/mol against BRAF^V600E^ and EGFR, respectively (Table 5). Observably, compound **3c** established one H-bond with the CYS773 (2.15 Å) of EGFR and two H-bonds with the LYS483 (2.13 Å) and ASP593 (1.93 Å) of BRAF^V600E^.

Erlotinib, a reference drug, showed docking scores of −8.4 and −8.6 kcal/mol toward BRAFV^600E^ and EGFR, respectively (Table 5). From Table 5, erlotinib demonstrated two H-bonds with the CYS773 (1.91 Å) and MET769 (1.62 Å) within the EGFR binding pocket. In addition, erlotinib also displayed two H-bonds with CYS532 (2.02 Å) and THR529 (2.07 Å).

## 3. Material and Methods

### 3.1. Chemistry

General information: refer to Supplementary Information.

The starting materials, **1a**–**i**, were synthesized in accordance with the documented methods [46,47,48]

#### 3.1.1. General Procedure of Synthesis of Trisubstituted Thiazoles **3a**–**i**

##### Method A

In a conical flask containing 10 mL ethyl acetate and two drops of Et_3_N as a catalyst, 0.092 gm of chloroacetone were dissolved (**2**). To this mixture, 1 mmol of thiosemicarbazides **1a**–**i** in 10 mL ethyl acetate was added drop by drop while stirring. After addition was complete, the reaction mixture was stirred for 24 h. The reaction mixture was monitored with TLC. After the reaction was completed, the formed precipitate was filtered off and recrystallized from ethanol to afford products **3a**–**i** as fine crystals.

##### Method B

In a 50 mL round-bottom flask containing 20 mL absolute ethanol, a molar ratio (1:1) mixture of chloroacetone and substituted thiosemicarbazides **1a**–**i** was added. The flask was fitted with a condenser and was refluxed for 6–10 h. The reaction was monitored with TLC to assure the reaction completion. Then, the reaction mixture was cooled to room temperature, and the formed precipitate was filtered off and recrystallized from ethanol to afford products **3a**–**i**.

##### (Z)-3-Benzyl-2-(2-(2,4-Dinitrophenyl)Hydrazineylidene)-4-Methyl-2,3-Dihydrothiazole (**3a**)

This compound was found as red crystals from methanol in (98% and 85%) yield, with m.p., 215–217 °C; ^1^H NMR (DMSO-*d_6_*): δ 1.98 (s, 3H, CH_3_), 5.00 (s, 2H, benzyl-CH_2_), 6.42 (s, 1H, H-5), 7.10–8.28 (m, 6H, Ar-H), 7.96–8.01 (d, 1H, Ar-H), 8.68 (s, 1H, Ar-H), 10.38 (s, 1H, NH) ppm; ^13^C NMR (DMSO-*d_6_*): δ 13.7 (CH_3_), 47.1 (benzyl-CH_2_), 94.5 (C5), 115.5, 123.4, 126.6, 127.4, 127.7, 128.7 (Ar-CH), 129.5, 136.7, 136.7, 144.3 (Ar-C), 135.1 (C4), 164.1 (C2) ppm; IR: ν = 3286 (NH),3085 (Ar-CH), 2978 (ali-CH), 1609 (C=N), 1548 (Ar-C=C), 1399, 1117 (NO_2_) cm^−1^; MS (70 eV): *m/z* (%) = 385 (M^+^, 30). *Anal. Calcd. For* C_17_H_15_N_5_O_4_S (385.40): C, 52.98; H, 3.92; N, 18.17; S, 8.32. Found: C, 52.89; H, 3.81; N, 18.06; S, 8.23.

##### (Z)-2-(2-(2,4-Dinitrophenyl)Hydrazineylidene)-3-Ethyl-4-Methyl-2,3-Dihydrothiazole (**3b**)

This compound was found as red crystals from methanol in (99% and 84%) yield, with m.p., 176–177 °C; ^1^H NMR (DMSO-*d_6_*): δ 1.28–1.38 (t, 3H, *J* = 3, CH_3_), 2.1 (s, 3H, CH_3_), 3.88–3.98 (q, 2H, *J* = 3, CH_2_), 6.14 (s, 1H, H-5), 7.48–7.54 (d, 1H, Ar-H), 8.22–8.28 (d, 1H, Ar-H), 8.82 (s, 1H, Ar-H), 10.46 (s, 1H, NH) ppm; ^13^C NMR (DMSO-*d_6_*): δ 12.9 (ethyl-CH_3_), 13.4 (CH_3_), 30.6 (ethyl-CH_2_), 94.0 (C5), 115.5, 123.4, 127.6 (Ar-CH), 129.7, 136.8, 144.3 (Ar-C), 135.0 (C4), 163.6 (C2) ppm. IR: ν = 3115 (NH), 3078 (Ar-CH), 2988 (ali-CH), 1612 (C=N), 1557 (Ar-C=C), 1373, 1132 (NO_2_). MS (70 eV): *m/z* (%) = 323 (M^+^, 54). *Anal. Calcd. For* C_12_H_13_N_5_O_4_S (323.33): C, 44.58; H, 4.05; N, 21.66; S, 9.92. Found: C, 44.46; H, 3.98; N, 21.57; S, 9.87.

##### (Z)-3-Allyl-2-(2-(2,4-Dinitrophenyl)Hydrazineylidene)-4-Methyl-2,3-Dihydrothiazole (**3c**)

This compound was found as red crystals from methanol in (96% and 83%) yield, with m.p. 197–198 °C; ^1^H NMR (DMSO-*d_6_*): δ 2.16 (s, 3H, CH_3_), 4.52–4.58 (m, 2H, allyl-CH_2_), 5.10–5.28 (m, 2H, allyl-CH_2_=), 5.96–6.06 (m, 1H, allyl-CH=), 6.16 (s, 1H, H-5), 7.46–7.52 (d, 1H, Ar-H), 8.22–8.28 (d, 1H, Ar-H), 8.85 (s, 1H, Ar-H), 10.48 (s, 1H, NH) ppm; ^13^C NMR (DMSO-*d_6_*): δ 13.4 (CH_3_), 46.1 (allyl-CH_2_), 94.17 (C5), 116.6 (allyl-CH_2_=), 115.6, 123.4, 128.3 (Ar-CH), 129.7, 136.9, 144.3 (Ar-C), 135.2 (C4), 136.9 (allyl-CH=), 164.4 (C2) ppm. IR: ν = 3105 (NH),3093 (Ar-CH), 2978 (ali-CH), 1606 (C=N), 1562 (Ar-C=C), 1374, 1206 (NO_2_). MS (70 eV): *m/z* (%) = 335 (M^+^, 93). *Anal. Calcd. For* C_13_H_13_N_5_O_4_S (335.34): C, 46.56; H, 3.91; N, 20.88; S, 9.56. Found: C, 46.48; H, 3.85; N, 20.79; S, 9.47**.**

##### (Z)-3-Cyclohexyl-2-(2-(2,4-Dinitrophenyl)Hydrazineylidene)-4-Methyl-2,3-Dihydrothi-Azole (**3d**)

This compound was found as red crystals from methanol in (94% and 78%) yield, with m.p., 204–206 °C; ^1^H NMR (DMSO-*d_6_*): δ 1.34–1.46 (m, 10H, cyclohexyl-CH_2_), 1.69–184 (m, 1H, cyclohexyl-CH), 2.21 (s, 3H, CH_3_), 6.10 (s, 1H, H-5), 7.36–7.39 (d, 1H, Ar-H), 8.29–8.32 (dd, 1H, Ar-H), 8.84–8.85 (d, 1H, Ar-H), 10.48 (s, 1H, NH) ppm. IR: ν = 3110 (NH),3015 (Ar-CH), 2925 (ali-CH), 1608 (C=N), 1544 (Ar-C=C), 1323, 1034 (NO_2_). MS (70 eV): *m/z* (%) = 377 (M^+^, 80) *Anal. Calcd. For* C_16_H_19_N_5_O_4_S (377.42): C, 50.92; H, 5.07; N, 18.56; S, 8.49. Found: C, 50.82; H, 4.93; N, 18.48; S, 8.43.

##### (Z)-2-(2-(2,4-Dinitrophenyl)Hydrazineylidene)-4-Methyl-3-Phenyl-2,3-Dihydrothiazole (**3e**)

This compound was found as red crystals from methanol in (94% and 83%) yield, with m.p., 231–233 °C; ^1^H NMR (DMSO-*d_6_*): δ 1.80 (s, 3H, CH_3_), 6.34 (s, 1H, H-5), 7.14–7.17 (d, 1H, Ar-H), 7.49–7.36 (m, 5H, Ar-H), 8.26–8.30 (dd, 1H, Ar-H), 8.85–8.86 (d, 1H, Ar-H), 10.47 (s, 1H, NH) ppm; ^13^C NMR (DMSO-*d_6_*): δ 14.5 (CH_3_), 95.3 (C5), 115.5, 123.3, 127.8, 128.5, 128.9, 129.6 (Ar-CH), 129.7, 136.2, 136.5, 144.5 (Ar-C), 135.2 (C4), 165.5 (C2) ppm; IR: ν = 3226 (NH), 3118 (Ar-CH), 2975 (ali-CH), 1603 (C=N),1555 (Ar-C=C), 1355, 1133 (NO_2_) cm^−1^; MS (70 eV): *m/z* (%) = 371 (M^+^, 8). *Anal. Calcd. For* C_16_H_13_N_5_O_4_S (371.37): C, 51.75; H, 3.53; N, 18.86; S, 8.63. Found: C, 51.68; H, 3.49; N, 18.79; S, 8.52.

##### (Z)-2-(2-(2,4-Dinitrophenyl)Hydrazineylidene)-4-Methyl-3-(p-Tolyl)-2,3-Dihydrothi- Azole (**3f**)

This compound was found as red crystals from methanol in (92% and 86%) yield, with m.p., 198–199 °C; ^1^H NMR (DMSO-*d_6_*): δ 1.87 (s, 3H, CH_3_), 2.40 (s, 3H, tolyl-CH_3_), 6.32 (s, 1H, H-5), 7.14–7.17 (d, 1H, Ar-H), 7.35–7.39 (m, 4H, Ar-H), 8.19–8.24 (dd, 1H, Ar-H), 8.81–8.83 (d, 1H, Ar-H), 10.47 (s, 1H, NH) ppm; ^13^C NMR (DMSO-*d_6_*): δ 14.5 (CH_3_), 20.7 (tolyl-CH_3_), 95.1 (C5), 115.5, 123.6, 127.8, 128.2, 129.7 (Ar-CH), 130.1, 134.1, 137.5, 138.4, 144.4 (Ar-C), 136.1 (C4), 165.4 (C2) ppm. IR: ν = 3226 (NH),3088 (Ar-CH), 2945 (ali-CH), 1607 (C=N), 1559 (Ar-C=C), 1383, 1172 (NO_2_). MS (70 eV): *m/z* (%) = 385 (M^+^, 13). *Anal. Calcd. For* C_17_H_15_N_5_O_4_S (385.40): C, 52.98; H, 3.92; N, 18.17; S, 8.32. Found: C, 52.89; H, 3.87; N, 18.11; S, 8.25.

##### (Z)-4-Methyl-N′-(4-Methyl-3-Phenylthiazol-2(3H)-Ylidene) Benzenesulfonohydrazide (**3g**)

This compound was found as pale-yellow crystals from methanol in (90% and 83%) yield, with m.p., 193–194 °C; ^1^H NMR (DMSO-*d_6_*): δ 2.13 (s, 3H, CH_3_), 2.30 (s, 3H, tolyl-CH_3_), 5.93 (s, 1H, H-5), 6.40 (d, 2H, tolyl-H-*m*), 6.93 (m, 1H, Ar-H), 7.19 (m, 2H, Ar-H), 7.31 (d, 2H, tolyl-H-*o*), 7.69 (m, 2H, Ar-H), 10.92 (s, 1H, NH) ppm; ^13^C NMR (DMSO-*d_6_*): δ 13.8 (CH_3_), 20.9 (tolyl-CH_3_), 91.0 (C5), 120.5, 122.9, 127.7, 128.9, 129.2 (Ar-CH), 137.6, 143.5, 148.5 (Ar-C), 136.0 (C4), 155.2 (C2) ppm. IR: ν = 3130 (NH),3062 (Ar-CH), 2920 (ali-CH), 1595 (C=N), 1561 (Ar-C=C). MS (70 eV): *m/z* (%) = 359 (M^+^, 100). *Anal. Calcd. For* C_17_H_17_N_3_O_2_S_2_ (359.46): C, 56.80; H, 4.77; N, 11.69; S, 17.84. Found: C, 56.73; H, 4.69; N, 11.63; S, 17.73.

##### (Z)-N′-(3-Allyl-4-Methylthiazol-2(3H)-Ylidene)-4-Methyl Benzenesulfonohydrazide (**3h**)

This compound was found as pale-yellow crystals from methanol in (89% and 83%) yield, with m.p., 236–238 °C. IR: ν = 3115 (NH),3078 (Ar-CH), 2988 (ali-CH), 1612 (C=N), 1557 (Ar-C=C), 1373, 1132 (NO_2_) cm^−1^. MS (70 eV): *m/z* (%) = 323 (M^+^, 100). *Anal. Calcd. For* C_14_H_17_N_3_O_2_S_2_ (323.43): C, 51.99; H, 5.30; N, 12.99; S, 19.83. Found: C, 51.91; H, 5.23; N, 12.92; S, 19.78.

##### (Z)-N′-(3-Ethyl-4-Methylthiazol-2(3H)-Ylidene)-4-Methyl Benzenesulfonohydrazide (**3i**)

This compound was found as pale-yellow crystals from methanol in (87% and 78%) yield, with m.p., 236–238 °C. ^1^H NMR (DMSO-*d_6_*): δ 1.26–1.39 (t, 3H, CH_3_), 2.12 (s, 3H, CH_3_), 3.80–3.96 (q, 2H, CH_2_), 5.91 (s, 1H, H-5), 6.42 (d, 2H, Ar-H), 7.13 (d, 2H, Ar-H), 10.89 ppm (s, 1H, NH). IR: ν = 3112 (NH), 3044 (Ar-CH), 2946 (ali-CH), 1613 (C=N), 1545 (Ar-C=C), cm^−1^;. MS (70 eV): *m/z* (%) = 311 (M^+^, 65). *Anal. Calcd. For* C_13_H_17_N_3_O_2_S_2_ (311.42): C, 50.14; H, 5.50; N, 13.49; O, 10.27; S, 20.59. Found: C, 50.09; H, 5.41; N, 13.39; S, 20.53.

#### 3.1.2. Crystal X-ray Structure Determination of **3b**

Compound **3b** was obtained as single crystals by recrystallization from methanol. Bruker D8 Venture diffractometer with Photon II detector at 298(2) K using Cu-Kα radiation (*λ* = 1.54178 Å) was used to study the single-crystal X-ray diffraction. Moreover, we used dual space methods (SHELXT for **5a**) [57,58] for the structure solution, and refinement was carried out using SHELXL-2014 (full-matrix least-squares on *F^2^*) [59]. Hydrogen atoms were localized by difference electron density determination and refined using a riding model. Semi-empirical absorption corrections and a general RIGU restraint were applied.

**3b**: red crystals, C_12_H_13_N_5_O_4_S, *M*_r_ = 323.33, crystal size 0.20 × 0.04 × 0.02 mm, triclinic, space group *P-1* (No. 2), *a* = 7.0981(2) Å, *b* = 8.2929(2) Å, *c* = 13.0081(4) Å, *α* = 101.598(1)°, *β* = 103.030(1)°, *γ* = 92.366(1)°, *V* = 727.74(4) Å^3^, *Z* = 2, *ρ* = 1.476 Mg/m^−3^, *µ*(Cu-K_α_) = 2.24 mm^−1^, *F*(000) = 336, *T* = 298 K, *2θ*_max_ = 144.4°, 13,774 reflections, of which 2874 were independent (*R*_int_ = 0.058), 200 parameters, 165 restraints (see cif-file for details), *R*_1_ = 0.063 (for 2692 I > 2σ(I)), w*R*_2_ = 0.170 (all data), *S* = 1.07, largest diff. peak/hole = 0.78/−0.40 e Å^−3^.

CCDC 2265616 (**3b**) contains the supplementary crystallographic data for this paper. These data can be obtained free of charge from the Cambridge Crystallographic Data Centre via www.ccdc.cam.ac.uk/data_request/cif (accessed on 24 June 2023).

### 3.2. Biology

#### 3.2.1. Cell Viability Assay

The human mammary gland epithelial (MCF-10A) cell line was used to test the viability of compounds **3a**–**i** [50,60]. See Supplementary Information.

#### 3.2.2. Antiproliferative Assay

The MTT assay was used to investigate **3a**–**i**’s antiproliferative activity versus four human cancer cell lines: colon cancer (HT-29) cell line, pancreatic cancer (Panc-1) cell line, lung cancer (A-549) cell line, and breast cancer (MCF-7) cell line, using erlotinib as the reference [52,53]. See Supplementary Information.

#### 3.2.3. EGFR Inhibitory Assay

Compounds **3a**, **3c**, **3d**, and **3f** were further evaluated for their suppressive effect versus EGFR as a probable molecular target for their mechanism of action [50,54]. See Supplementary Information.

#### 3.2.4. BRAF^V600E^ Inhibitory Assay

Derivatives **3a**, **3c**, **3d**, and **3f** were further investigated as possible BRAF^V600E^ inhibitors [61]. See Supplementary Information.

### 3.3. In Silico Study

The crystal structures of BRAF^V600E^ and EGFR, with PDB codes 3OG7 [62] and 1M17 [63], respectively, were prepared for all docking computations. All heteroatoms, water molecules, ligands, and ions were removed to prepare the PDB files. Modeler software was applied to construct all missing amino acids [64,65]. The protonation state of titratable residues of the investigated targets was estimated using PropKa software at pH 7.0 [66]. The 3D structure of the investigated compounds was energetically minimized using the MMFF94S force field within SZYBKI software [67,68].

For docking computations, AutoDock4.2.6 software was utilized [69]. All docking parameters were set to default values, except *GA* run and energy evaluation, which were 250 and 25,000,000, respectively. The active site of the investigated targets was inspected by a grid box with a size of 50 Å × 50 Å × 50 Å. The grid maps were generated using the AutoGrid program with a spacing of 0.375 Å. Gasteiger–Marsili method was employed to assign the atomic charges of the chemical compounds [70]. Discovery Studio module of Biovia software 17.1.0.115 was utilized to visualize all drug–protein interactions [71].

## 4. Conclusions

Using simple interactions between thiosemicarbazides and chloroacetone, a novel set of heterocycles with thiazole rings was developed. All obtained derivatives were validated using various spectral data such as IR, NMR, mass spectrometry, elemental analysis, and X-ray crystallography. The newly synthesized compounds, **3a**–**i**, were evaluated against a panel of four human cancer cell lines, with compounds **3a**, **3c**, **3d**, and **3f** being the most potent variants. The in vitro assay results demonstrated that compound 3f possesses potent antiproliferative activity as a dual EGFR/BRAF^V600E^ inhibitor, signaling that further structural modifications may be needed to establish a more potent lead molecule for future development. Finally, the docking analysis results showed that all inspected compounds revealed good docking scores toward BRAF^V600E^ and EGFR.

## Figures and Tables

**Scheme 1:**
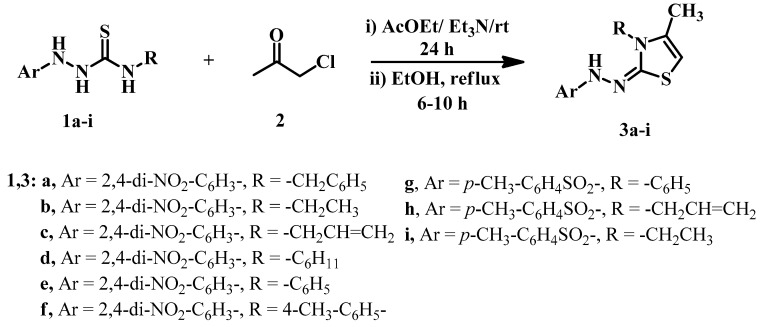
Syntheses of substituted thiazoles **3a**–**i**.

**Table 1:** Yield percentage of **3a**–**i** via method **A** and method **B**.

Compound	3a	3b	3c	3d	3e	3f	3g	3h	3i
Method A	98	99	96	94	94	92	90	89	87
Method B	85	84	83	78	83	86	83	83	78

**Table 2:** Selected geometric parameters (Å, °) for **3b**.

S1—C5	1.742 (2)	N3—C4	1.394 (2)
S1—C2	1.7465 (19)	C4—C5	1.327 (3)
C2—N6	1.298 (2)	N6—N7	1.377 (2)
C2—N3	1.370 (2)	N7—C8	1.337 (2)
C5—S1—C2	90.45 (9)	N3—C4—C22	119.73 (19)
N6—C2—N3	121.44 (17)	C4—C5—S1	112.19 (14)
N6—C2—S1	128.53 (15)	C4—C5—H5	123.9
N3—C2—S1	110.03 (13)	S1—C5—H5	123.9
C2—N3—C4	113.80 (16)	C2—N6—N7	113.44 (16)
C2—N3—C20	120.49 (17)	C8—N7—N6	121.10 (17)
C4—N3—C20	125.43 (18)	C8—N7—H7	119.5
C5—C4—N3	113.50 (17)	N6—N7—H7	119.5
C5—C4—C22	126.76 (19)		
N3—C2—N6—N7	−179.55 (16)	N6—N7—C8—C9	1.6 (3)
S1—C2—N6—N7	0.1 (3)	N6—N7—C8—C13	−178.62 (16)
C2—N6—N7—C8	−174.03 (17)		

**Table 3:** IC_50_ values of compounds **3a–i** against four cancer cell lines.

Compound	Cell Viability %	Antiproliferative Activity IC_50_ ± SEM (nM)				
		A-549	MCF-7	Panc-1	HT-29	Average
**3a**	89	45 ± 4	49 ± 4	48 ± 4	48 ± 4	48
**3b**	91	78 ± 7	82 ± 8	80 ± 8	80 ± 8	80
**3c**	90	50 ± 5	56 ± 5	54± 5	54 ± 5	54
**3d**	92	43 ± 4	47 ± 4	46 ± 4	46 ± 4	46
**3e**	87	71 ± 7	74 ± 7	72 ± 7	72 ± 7	72
**3f**	89	35 ± 3	40 ± 3	37 ± 3	37 ± 3	37
**3g**	90	58 ± 5	63 ± 6	60± 6	58 ± 5	60
**3h**	92	63 ± 6	68 ± 6	65 ± 6	65 ± 6	65
**3i**	95	84 ± 8	89 ± 8	86 ± 8	86 ± 8	86
**Erlotinib**	-	30 ± 3	40 ± 3	30 ± 3	30 ± 3	33

**Table 4:** IC_50_ of compounds **3a**, **3c**, **3d**, and **3f** against EGFR and BRAF^V600E^.

Compound	EGFR Inhibition IC_50_ ± SEM (nM)	BRAF^V600E^ Inhibition IC_50_ ± SEM (nM)
**3a**	93 ± 8	117 ± 10
**3c**	98 ± 9	126 ± 11
**3d**	91 ± 7	112 ± 10
**3f**	89 ± 7	93 ± 8
**Erlotinib**	80 ± 5	60 ± 5

**Table 5:** Predicted binding features and docking scores for the top investigated compounds toward EGFR and BRAF^V600E^.

Compound	EGFR		BRAF^V600E^	
	Docking Scores (kcal/mol)	Binding Features	Docking Scores (kcal/mol)	Binding Features ^a^
**3a**	−8.0	CYS773 (2.21 Å)	−8.4	LYS483 (2.15 Å), THR529 (2.38 Å), ASP594 (2.12 Å)
**3c**	−7.9	CYS773 (2.15 Å)	−7.8	LYS483 (2.13 Å) ASP594 (1.93 Å)
**3d**	−8.1	CYS773 (2.17 Å)	−8.5	LYS483 (1.93 Å), GLY596 (2.32 Å), THR529 (2.66 Å), ASP594 (2.14 Å)
**3f**	−8.5	CYS773 (2.18 Å)	−8.7	LYS483 (2.15 Å), THR529 (2.38 Å), ASP594 (2.12 Å)
**Erlotinib**	−8.6	MET769 (1.62 Å), CYS773 (1.91 Å)	−8.4	THR529 (2.07), CYS532 (2.02)

^a^ Only hydrogen bonds are presented in Å.

## Data Availability

Data is contained within the article and Supplementary Material.

## Supplementary Materials

The following supporting information can be downloaded at https://www.mdpi.com/article/10.3390/ph16071014/s1. Figure S1: The crystal structure of (Z)-2-(2-(2,4-dinitrophenyl)hydrazineylidene)-3-ethyl-4-methyl-2,3-dihydrothiazole **3b**; Figures S2–S32: IR and NMR analysis of the structure assignment for all obtained products **3a**–**i**.
