# Supplementary material for: Synthesis and Structure Determination of Substituted Thiazole Derivatives as EGFR/BRAFV600E Dual Inhibitors Endowed with Antiproliferative Activity
Source: Pharmaceuticals (Basel). 2023 Jul 17;16(7):1014. doi: 10.3390/ph16071014 (PMC10384562; doi:10.3390/ph16071014)
Supplement: Supplementary file 1 [file pharmaceuticals-16-01014-s001.zip › pharmaceuticals-2476427-supplementary.pdf]

Supplementary Information

Synthesis and Structure Determination of Substituted Thiazole Derivatives as EGFR/BRAF^{V600E} Dual Inhibitors Endowed with Antiproliferative Activity

Lamya H. Al-Wahaibi [1,*], Essmat M. El-Sheref [2], Alaa A. Hassan [2], S. Bräse [3,*], M. Nieger [4], Bahaa G. M. Youssif [5,*], Mahmoud A. A. Ibrahim [2,6] and Hendawy N. Tawfeek [2,7]

1 Department of Chemistry, College of Sciences, Princess Nourah bint Abdulrahman University, Riyadh 11564, Saudi Arabia
2 Chemistry Department, Faculty of Science, Minia University, El Minia 61519, Egypt
3 Institute of Biological and Chemical Systems, IBCS-FMS, Karlsruhe Institute of Technology, 76131 Karlsruhe, Germany
4 Department of Chemistry, University of Helsinki, P.O. Box 55 (A. I. Virtasen aukio 1), 00014 Helsinki, Finland
5 Pharmaceutical Organic Chemistry Department, Faculty of Pharmacy, Assiut University, Assiut 71526, Egypt
6 School of Health Sciences, University of KwaZulu-Natal, Westville Campus, Durban 4000, South Africa
7 Unit of Occupational of Safety and Health, Administration Office of Minia University, El-Minia 61519, Egypt
* Correspondence: lhalwahaibi@pnu.edu.sa (L.H.A.-W.); stefan.braese@kit.edu (S.B.); bgyoussif2@gmail.com (B.G.M.Y.)

Crystal X-ray structure determination of compound 3b.

Compound **3b**: $C_{12}H_{13}N_5O_4S$, Mr = 323.33 g mol^{-1}, plates red crystal, size 0.20 × 0.04 × 0.02 mm, Triclinic, *P*-1 (no.2), a = 7.0981 (2) Å, b = 8.2929 (2) Å, c = 13.0081 (4) Å, V = 727.74 (4) Å^3, α = 101.598 (1)° β = 103.030 (1)°, γ = 92.366 (1)°, λ = 1.54178 Å, Z = 2, D_{calcd} = 1.476 Mg m^{-3}, *F*(000) = 336, μ = 2.24 mm^{-1}, T = 298 K, 13774 measured reflections ($2\theta_{max}$ = 144.4°,), 2874 independent [R_{int} = 0.058], 200 parameters, 165 restraints, R1 [for 2692 I > 2r(I)] = 0.063, wR2 (for all data) = 0.170, S = 1.07, largest diff. peak and hole = = 0.78 e Å^{-3}-0.40 e Å^{-3}. The absolute structure was determined by refinement of Parsons parameter x = 0.01(2) (Parsons, S., Flack, H. D. Acta Crystallogr. 2004, A60, s61).

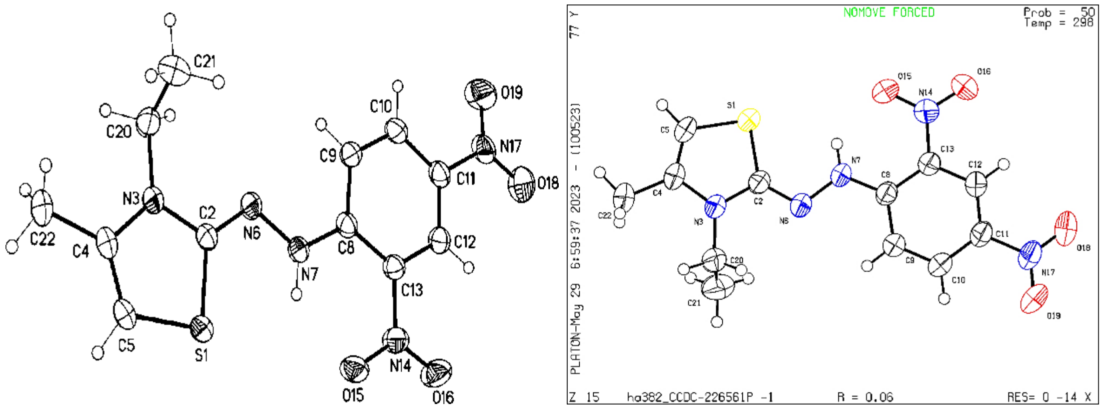

Figure S1. The crystal Structure of (Z)-2-(2-(2,4-dinitrophenyl)hydrazineylidene)-3-ethyl-4-methyl-2,3-dihydrothiazole **3b** (Thermal ellipsoids are shown at 50% probability level.).

Crystal data

$C_{12}H_{13}N_5O_4S$	Z = 2
M_r = 323.33	F(000) = 336
Triclinic, P-1 (no.2)	D_x = 1.476 Mg m^{-3}
a = 7.0981 (2) Å	Cu $K\alpha$ radiation, λ = 1.54178 Å
b = 8.2929 (2) Å	Cell parameters from 9276 reflections
c = 13.0081 (4) Å	θ = 3.5–72.1°
α = 101.598 (1)°	μ = 2.24 mm^{-1}
β = 103.030 (1)°	T = 298 K
γ = 92.366 (1)°	Plates, red
V = 727.74 (4) Å^3	0.20 × 0.04 × 0.02 mm

Data collection

Bruker D8 VENTURE diffractometer with PhotonII CPAD detector	2692 reflections with $I > 2\sigma(I)$
Radiation source: INCOATEC microfocus sealed tube	R_{int} = 0.058
rotation in ϕ and ω, 1°, shutterless scans	θ_{max} = 72.2°, θ_{min} = 3.6°
Absorption correction: multi-scan *SADABS* (Sheldrick, 2014)	h = -8→8
T_{min} = 0.621, T_{max} = 0.971	k = -10→10
13774 measured reflections	l = -16→15
2874 independent reflections	

Refinement

Refinement on F^2	Primary atom site location: dual
Least-squares matrix: full	Secondary atom site location: difference Fourier map
$R[F^2 > 2\sigma(F^2)] = 0.063$	Hydrogen site location: inferred from neighbouring sites
$wR(F^2) = 0.170$	H-atom parameters constrained
$S = 1.07$	$w = 1/[\sigma^2(F_o^2) + (0.125P)^2 + 0.1016P]$ where $P = (F_o^2 + 2F_c^2)/3$
2874 reflections	$(\Delta/\sigma)_{max} < 0.001$
200 parameters	$\Delta\rangle_{max} = 0.78$ e Å^{-3}
165 restraints	$\Delta\rangle_{min} = -0.40$ e Å^{-3}

Fractional atomic coordinates and isotropic or equivalent isotropic displacement parameters (Å^2) for (ha382)

	x	*y*	*z*	U_{iso}*/U_{eq}
S1	0.23022 (7)	0.63710 (5)	0.59362 (4)	0.0477 (2)
C2	0.2786 (3)	0.4299 (2)	0.57661 (15)	0.0429 (4)
N3	0.3037 (3)	0.3814 (2)	0.67314 (13)	0.0491 (4)
C4	0.2799 (3)	0.5049 (3)	0.75813 (15)	0.0502 (5)
C5	0.2428 (3)	0.6477 (2)	0.73013 (16)	0.0505 (5)
H5	0.2255	0.7425	0.7779	0.061*
N6	0.2924 (3)	0.3285 (2)	0.48913 (13)	0.0485 (4)
N7	0.2643 (2)	0.3987 (2)	0.40013 (13)	0.0453 (4)
H7	0.2516	0.5029	0.4080	0.054*
C8	0.2565 (2)	0.3077 (2)	0.30172 (14)	0.0421 (4)
C9	0.2725 (3)	0.1351 (2)	0.28701 (16)	0.0500 (5)
H9	0.2853	0.0858	0.3461	0.060*
C10	0.2693 (3)	0.0403 (3)	0.18830 (18)	0.0543 (5)
H10	0.2805	-0.0727	0.1804	0.065*
C11	0.2493 (3)	0.1128 (3)	0.09837 (16)	0.0509 (5)
C12	0.2286 (3)	0.2775 (3)	0.10739 (16)	0.0514 (5)
H12	0.2123	0.3237	0.0468	0.062*
C13	0.2319 (3)	0.3754 (2)	0.20769 (16)	0.0463 (4)
N14	0.2122 (3)	0.5477 (2)	0.21214 (16)	0.0598 (5)
O15	0.2115 (3)	0.64000 (19)	0.29996 (15)	0.0711 (5)
O16	0.1984 (4)	0.6007 (2)	0.12911 (17)	0.0906 (7)
N17	0.2508 (3)	0.0114 (3)	-0.00590 (15)	0.0656 (5)
O18	0.2206 (4)	0.0723 (3)	-0.08562 (15)	0.0981 (8)

O19	0.2831 (5)	-0.1312 (3)	-0.01113 (19)	0.1083 (9)
C20	0.3329 (4)	0.2088 (3)	0.67862 (19)	0.0638 (6)
H20A	0.4001	0.2045	0.7516	0.077*
H20B	0.4143	0.1658	0.6306	0.077*
C21	0.1444 (5)	0.1021 (3)	0.6475 (3)	0.0893 (9)
H21A	0.1696	-0.0094	0.6521	0.134*
H21B	0.0645	0.1430	0.6957	0.134*
H21C	0.0786	0.1044	0.5748	0.134*
C22	0.2996 (4)	0.4688 (3)	0.86815 (18)	0.0694 (7)
H22A	0.2850	0.5672	0.9180	0.104*
H22B	0.4254	0.4320	0.8919	0.104*
H22C	0.2011	0.3839	0.8651	0.104*

Atomic displacement parameters (Å^2) for (ha382)

	U^{11}	U^{22}	U^{33}	U^{12}	U^{13}	U^{23}
S1	0.0634 (3)	0.0383 (3)	0.0403 (3)	0.0079 (2)	0.0143 (2)	0.0033 (2)
C2	0.0494 (9)	0.0395 (9)	0.0371 (9)	0.0058 (7)	0.0095 (7)	0.0029 (7)
N3	0.0693 (10)	0.0413 (8)	0.0343 (8)	0.0095 (7)	0.0114 (6)	0.0030 (6)
C4	0.0610 (10)	0.0505 (11)	0.0341 (9)	0.0036 (8)	0.0105 (7)	-0.0011 (8)
C5	0.0643 (11)	0.0438 (10)	0.0379 (9)	0.0041 (8)	0.0132 (8)	-0.0047 (7)
N6	0.0660 (9)	0.0435 (8)	0.0363 (8)	0.0120 (7)	0.0150 (7)	0.0043 (6)
N7	0.0615 (9)	0.0399 (8)	0.0343 (8)	0.0089 (6)	0.0155 (6)	0.0023 (6)
C8	0.0470 (8)	0.0407 (9)	0.0377 (9)	0.0039 (7)	0.0135 (7)	0.0026 (7)
C9	0.0686 (11)	0.0410 (10)	0.0398 (10)	0.0057 (8)	0.0139 (8)	0.0063 (8)
C10	0.0751 (12)	0.0388 (9)	0.0458 (10)	0.0037 (8)	0.0155 (9)	0.0005 (8)
C11	0.0638 (11)	0.0461 (10)	0.0389 (10)	0.0000 (8)	0.0143 (8)	-0.0014 (8)
C12	0.0678 (12)	0.0490 (11)	0.0383 (9)	0.0019 (8)	0.0171 (8)	0.0074 (8)
C13	0.0586 (10)	0.0401 (9)	0.0420 (10)	0.0043 (7)	0.0180 (8)	0.0065 (7)
N14	0.0879 (12)	0.0442 (9)	0.0532 (10)	0.0110 (8)	0.0265 (9)	0.0124 (8)
O15	0.1155 (14)	0.0423 (8)	0.0592 (10)	0.0187 (8)	0.0314 (9)	0.0054 (7)
O16	0.167 (2)	0.0569 (10)	0.0622 (11)	0.0235 (11)	0.0433 (12)	0.0264 (9)
N17	0.0919 (14)	0.0558 (11)	0.0438 (10)	-0.0010 (9)	0.0211 (9)	-0.0054 (8)
O18	0.164 (2)	0.0869 (14)	0.0407 (9)	0.0124 (14)	0.0292 (11)	0.0018 (9)
O19	0.204 (3)	0.0550 (11)	0.0659 (13)	0.0184 (13)	0.0502 (15)	-0.0094 (9)
C20	0.0985 (16)	0.0477 (11)	0.0457 (11)	0.0226 (11)	0.0149 (10)	0.0103 (9)
C21	0.130 (2)	0.0464 (13)	0.0808 (19)	-0.0035 (14)	0.0119 (17)	0.0068 (12)
C22	0.1037 (18)	0.0669 (14)	0.0348 (10)	0.0100 (13)	0.0166 (10)	0.0040 (9)

Geometric parameters (Å, º) for (ha382)

S1—C5	1.742 (2)	C11—C12	1.365 (3)
S1—C2	1.7465 (19)	C11—N17	1.448 (3)
C2—N6	1.298 (2)	C12—C13	1.386 (3)
C2—N3	1.370 (2)	C12—H12	0.9300
N3—C4	1.394 (2)	C13—N14	1.432 (3)
N3—C20	1.468 (3)	N14—O16	1.231 (3)
C4—C5	1.327 (3)	N14—O15	1.242 (3)
C4—C22	1.498 (3)	N17—O19	1.206 (3)
C5—H5	0.9300	N17—O18	1.223 (3)
N6—N7	1.377 (2)	C20—C21	1.501 (5)
N7—C8	1.337 (2)	C20—H20A	0.9700
N7—H7	0.8600	C20—H20B	0.9700
C8—C9	1.418 (3)	C21—H21A	0.9600
C8—C13	1.425 (3)	C21—H21B	0.9600
C9—C10	1.359 (3)	C21—H21C	0.9600
C9—H9	0.9300	C22—H22A	0.9600
C10—C11	1.402 (3)	C22—H22B	0.9600
C10—H10	0.9300	C22—H22C	0.9600
C5—S1—C2	90.45 (9)	C11—C12—H12	120.3
N6—C2—N3	121.44 (17)	C13—C12—H12	120.3
N6—C2—S1	128.53 (15)	C12—C13—C8	121.40 (18)
N3—C2—S1	110.03 (13)	C12—C13—N14	116.71 (18)
C2—N3—C4	113.80 (16)	C8—C13—N14	121.89 (18)
C2—N3—C20	120.49 (17)	O16—N14—O15	121.77 (19)
C4—N3—C20	125.43 (18)	O16—N14—C13	119.16 (19)
C5—C4—N3	113.50 (17)	O15—N14—C13	119.07 (17)
C5—C4—C22	126.76 (19)	O19—N17—O18	122.0 (2)
N3—C4—C22	119.73 (19)	O19—N17—C11	118.5 (2)
C4—C5—S1	112.19 (14)	O18—N17—C11	119.5 (2)
C4—C5—H5	123.9	N3—C20—C21	112.0 (2)
S1—C5—H5	123.9	N3—C20—H20A	109.2
C2—N6—N7	113.44 (16)	C21—C20—H20A	109.2
C8—N7—N6	121.10 (17)	N3—C20—H20B	109.2
C8—N7—H7	119.5	C21—C20—H20B	109.2
N6—N7—H7	119.5	H20A—C20—H20B	107.9
N7—C8—C9	120.14 (18)	C20—C21—H21A	109.5
N7—C8—C13	123.12 (18)	C20—C21—H21B	109.5

C9—C8—C13	116.74 (17)	H21A—C21—H21B	109.5
C10—C9—C8	121.38 (19)	C20—C21—H21C	109.5
C10—C9—H9	119.3	H21A—C21—H21C	109.5
C8—C9—H9	119.3	H21B—C21—H21C	109.5
C9—C10—C11	119.95 (19)	C4—C22—H22A	109.5
C9—C10—H10	120.0	C4—C22—H22B	109.5
C11—C10—H10	120.0	H22A—C22—H22B	109.5
C12—C11—C10	121.13 (19)	C4—C22—H22C	109.5
C12—C11—N17	119.34 (19)	H22A—C22—H22C	109.5
C10—C11—N17	119.53 (19)	H22B—C22—H22C	109.5
C11—C12—C13	119.37 (19)		
C5—S1—C2—N6	179.58 (19)	C9—C10—C11—C12	-1.4 (3)
C5—S1—C2—N3	-0.78 (15)	C9—C10—C11—N17	178.5 (2)
N6—C2—N3—C4	-178.76 (17)	C10—C11—C12—C13	1.5 (3)
S1—C2—N3—C4	1.6 (2)	N17—C11—C12—C13	-178.43 (19)
N6—C2—N3—C20	-4.5 (3)	C11—C12—C13—C8	0.0 (3)
S1—C2—N3—C20	175.81 (17)	C11—C12—C13—N14	179.14 (19)
C2—N3—C4—C5	-1.8 (3)	N7—C8—C13—C12	178.65 (18)
C20—N3—C4—C5	-175.7 (2)	C9—C8—C13—C12	-1.5 (3)
C2—N3—C4—C22	178.9 (2)	N7—C8—C13—N14	-0.5 (3)
C20—N3—C4—C22	5.0 (3)	C9—C8—C13—N14	179.35 (18)
N3—C4—C5—S1	1.1 (2)	C12—C13—N14—O16	-1.8 (3)
C22—C4—C5—S1	-179.6 (2)	C8—C13—N14—O16	177.4 (2)
C2—S1—C5—C4	-0.20 (17)	C12—C13—N14—O15	179.0 (2)
N3—C2—N6—N7	-179.55 (16)	C8—C13—N14—O15	-1.9 (3)
S1—C2—N6—N7	0.1 (3)	C12—C11—N17—O19	174.8 (3)
C2—N6—N7—C8	-174.03 (17)	C10—C11—N17—O19	-5.2 (4)
N6—N7—C8—C9	1.6 (3)	C12—C11—N17—O18	-4.8 (4)
N6—N7—C8—C13	-178.62 (16)	C10—C11—N17—O18	175.2 (2)
N7—C8—C9—C10	-178.53 (19)	C2—N3—C20—C21	-82.6 (3)
C13—C8—C9—C10	1.6 (3)	C4—N3—C20—C21	90.9 (3)
C8—C9—C10—C11	-0.2 (3)		

Hydrogen-bond geometry (Å, º) for (ha382)

D—H···*A*	*D*—H	H···*A*	*D*···*A*	*D*—H···*A*
N7—H7···S1	0.86	2.49	2.9351 (16)	113
N7—H7···O15	0.86	1.96	2.595 (2)	130

Document origin: *publCIF* [Westrip, S. P. (2010). *J. Apply. Cryst.*, **43**, 920-925].

Spectral data

Spectral data of compound **3a**:

Figure S2: IR spectra of **3a**

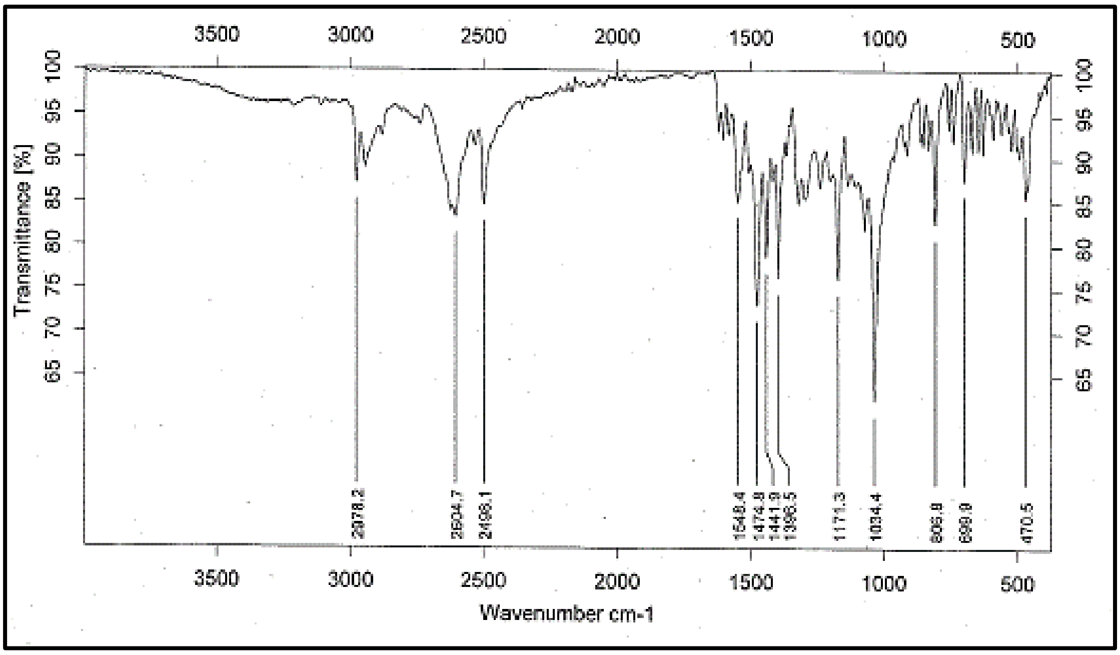

Figure S3: Mass spectrum of **3a**

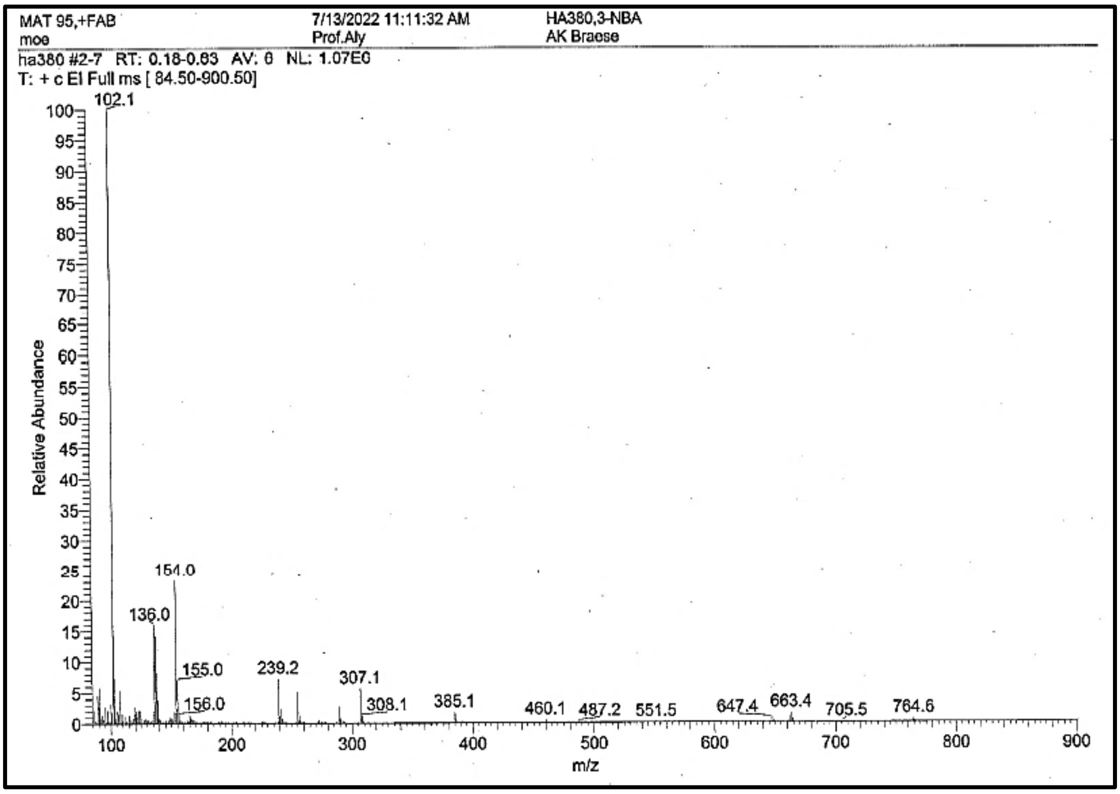

Figure S4: ^{1}H NMR of **3a**

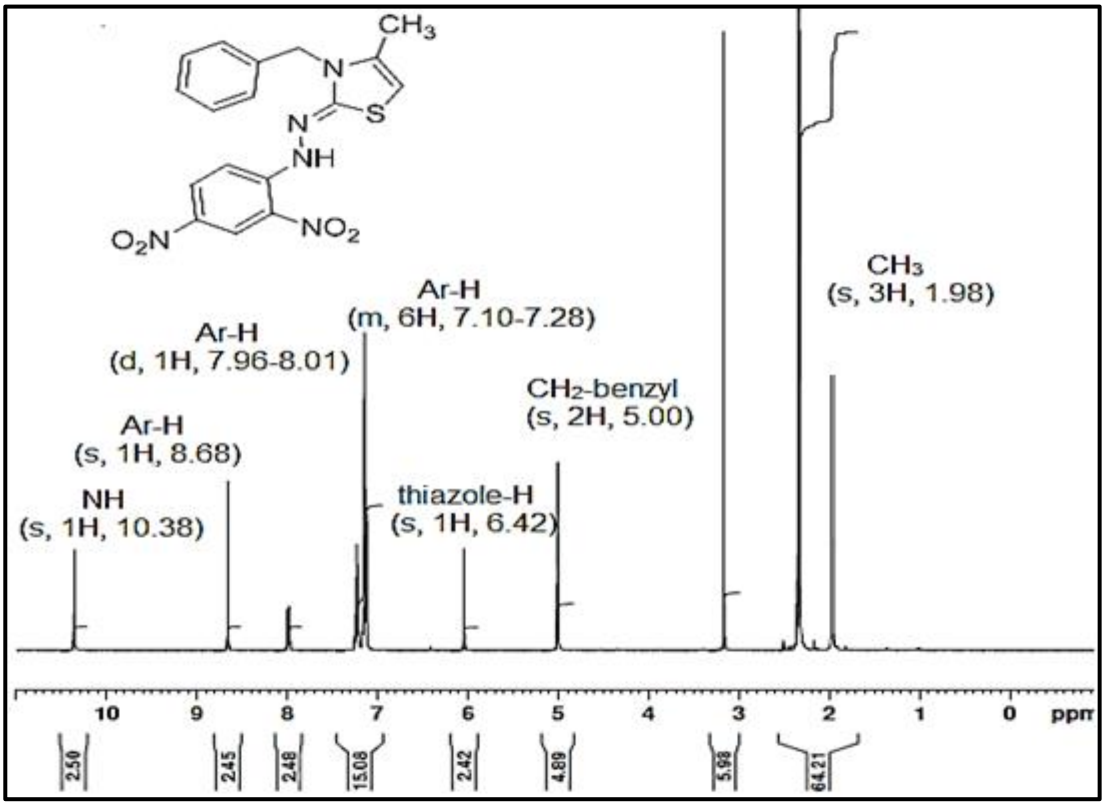

Figure S5: ^{13}C NMR of **3a**

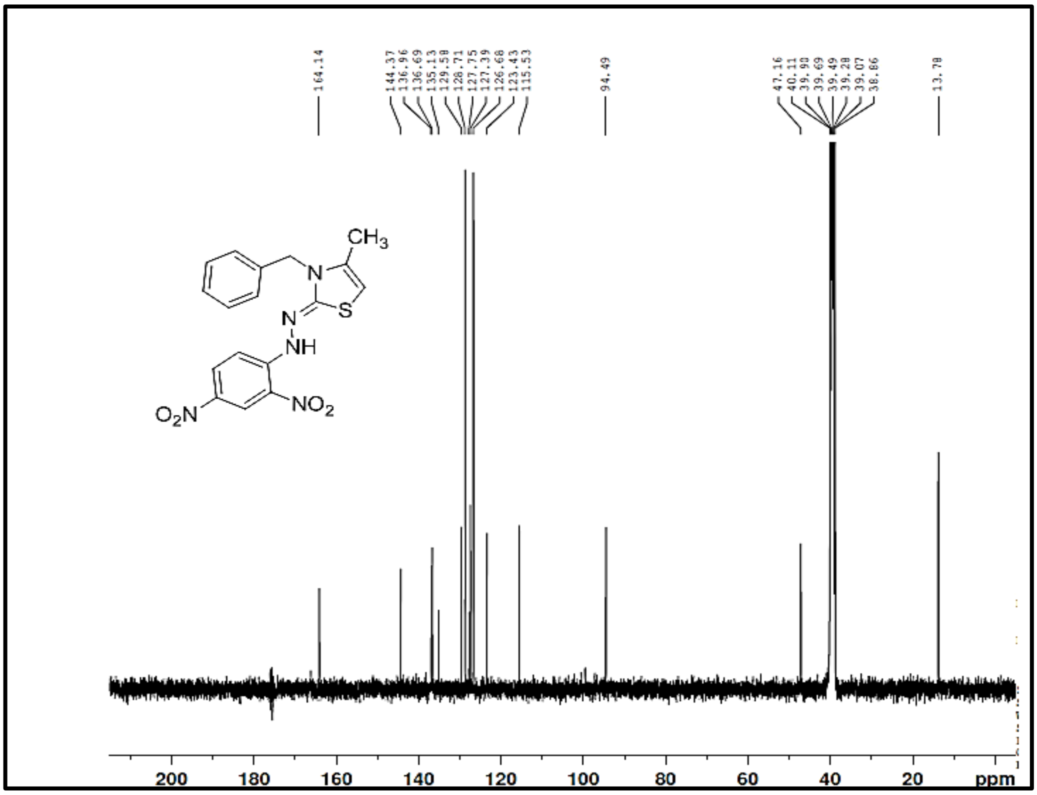

Spectral data of compound **3b**:

Figure S6: IR spectrum of **3b**

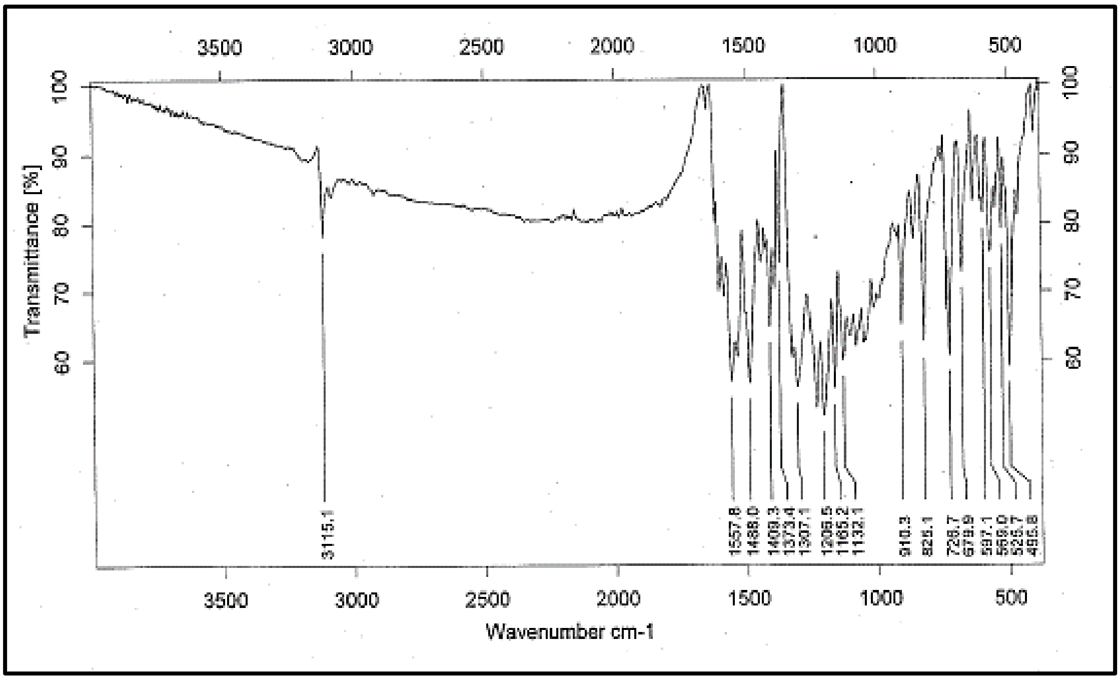

Figure S7: Mass spectrum of **3b**

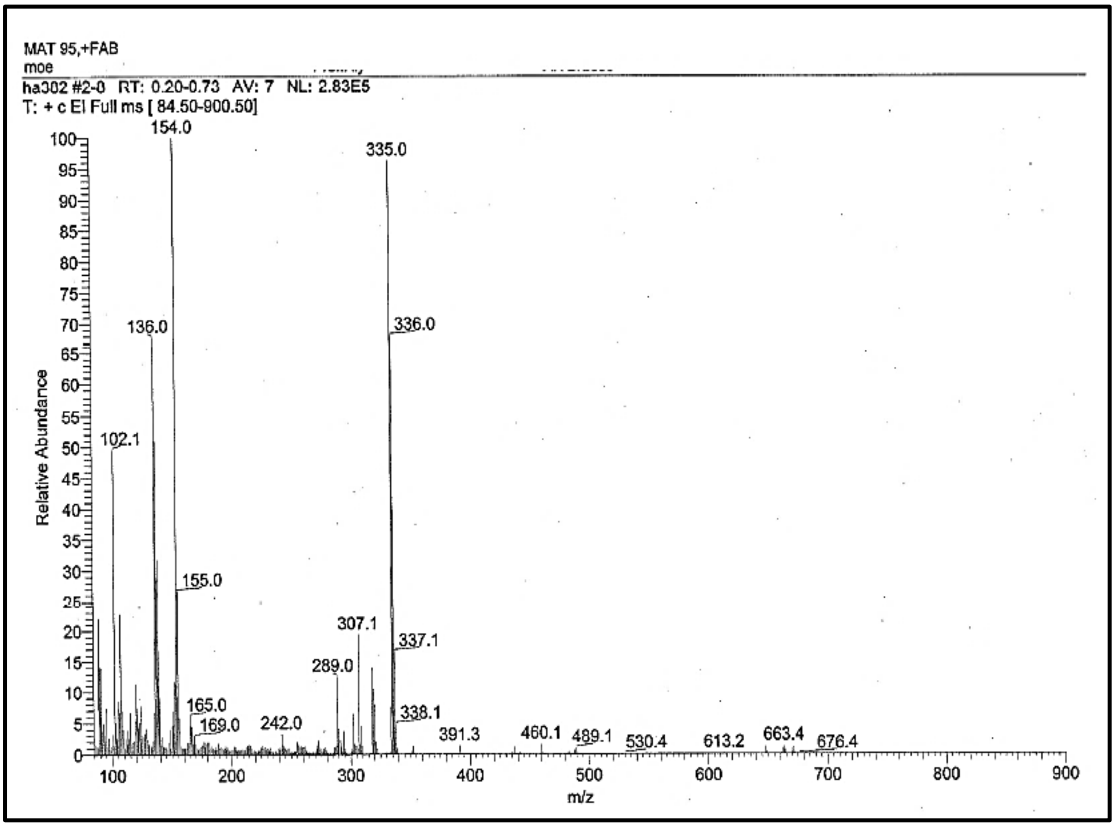

Figure S8: ^{1}H NMR of **3b**

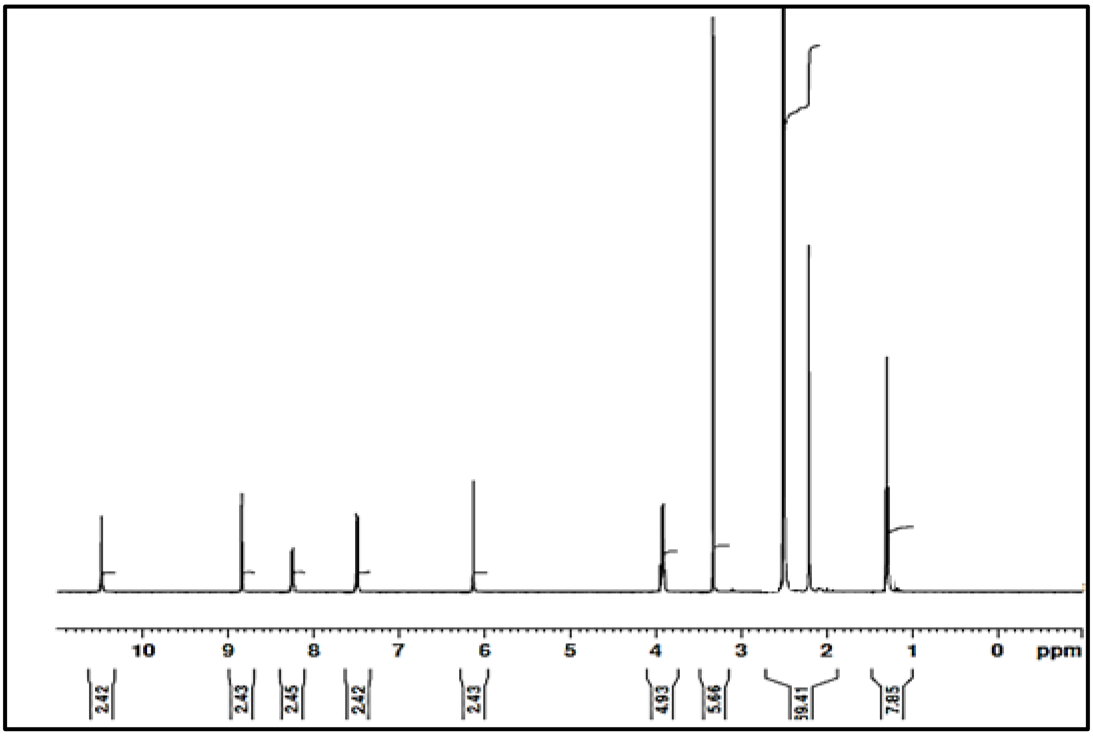

Figure S9: ^{13}C NMR of **3b**

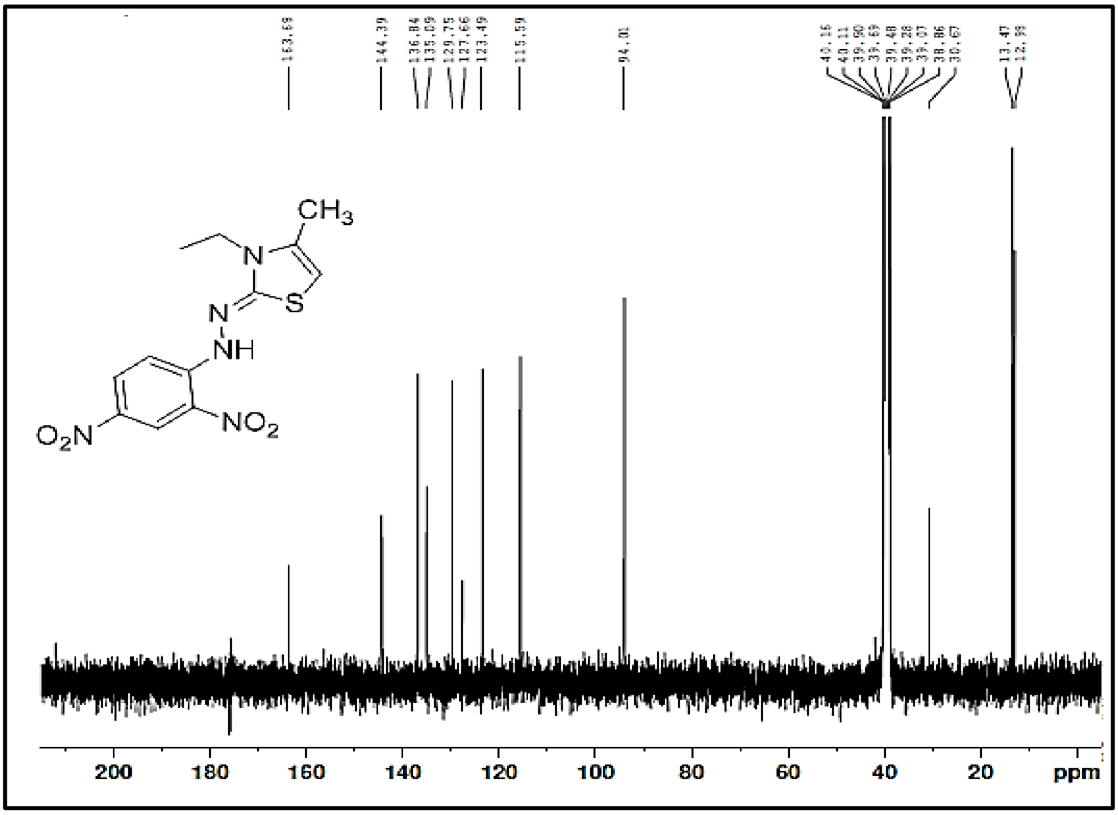

Spectral data of compound **3c**:

Figure S10: IR spectrum of **3c**

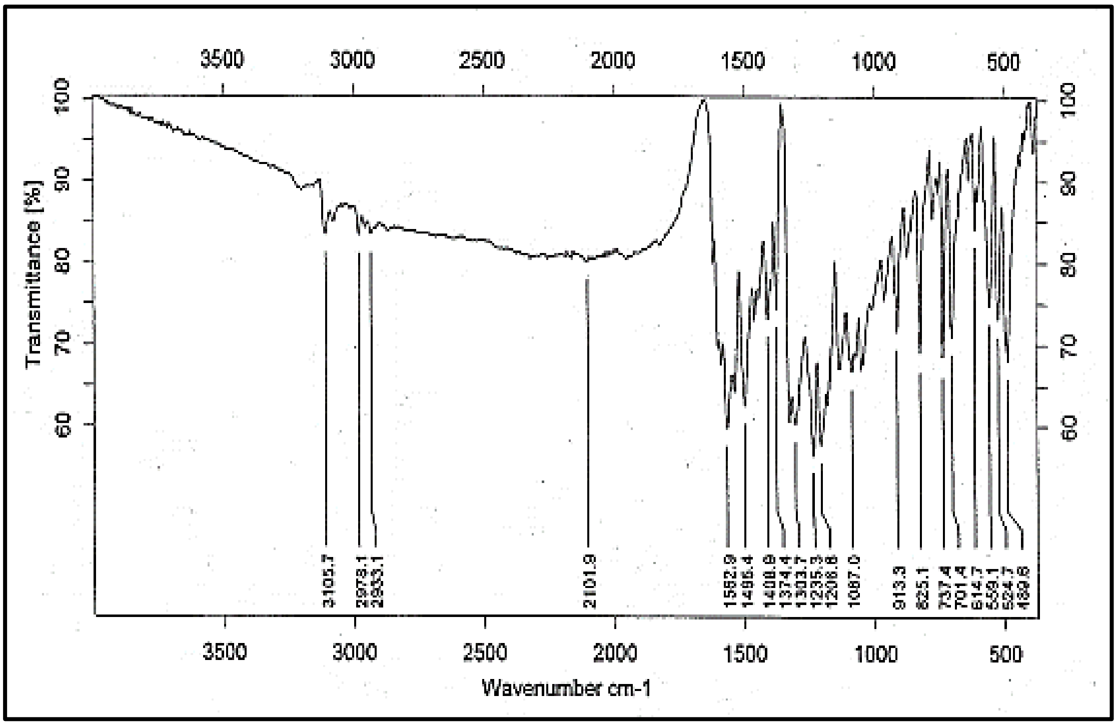

Figure S11: Mass spectrum of **3c**

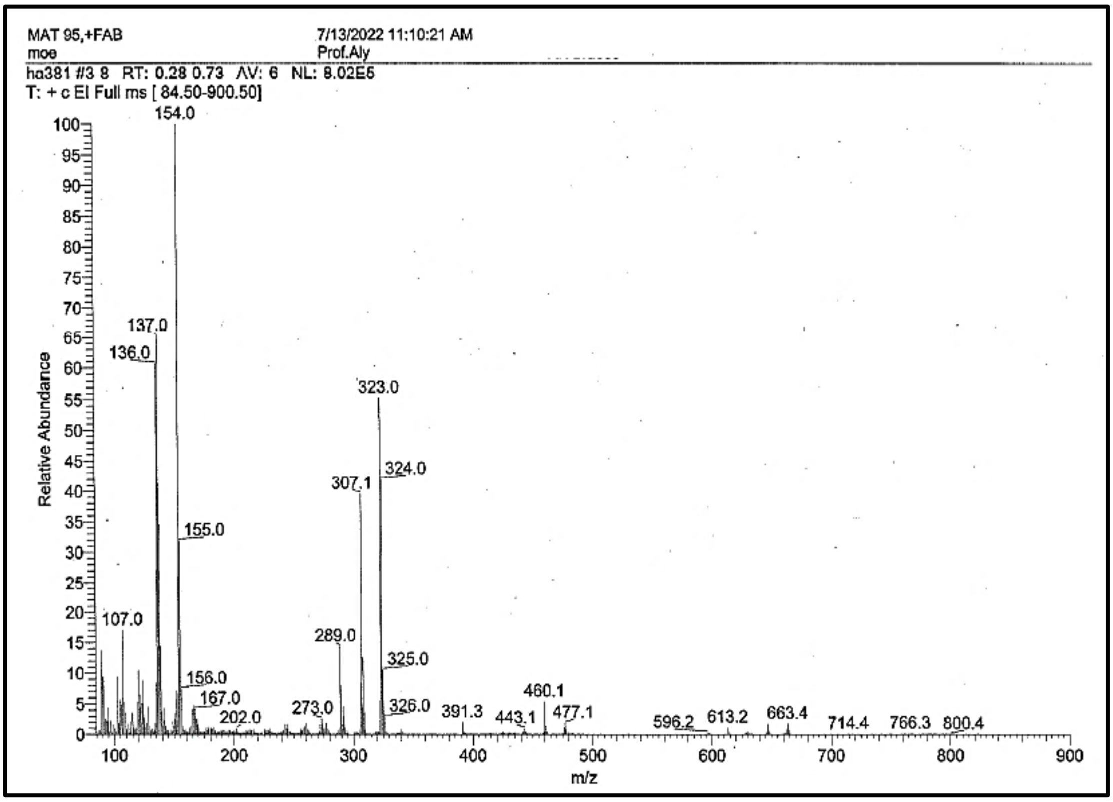

Figure S12: ^{1}H NMR spectrum of **3c**

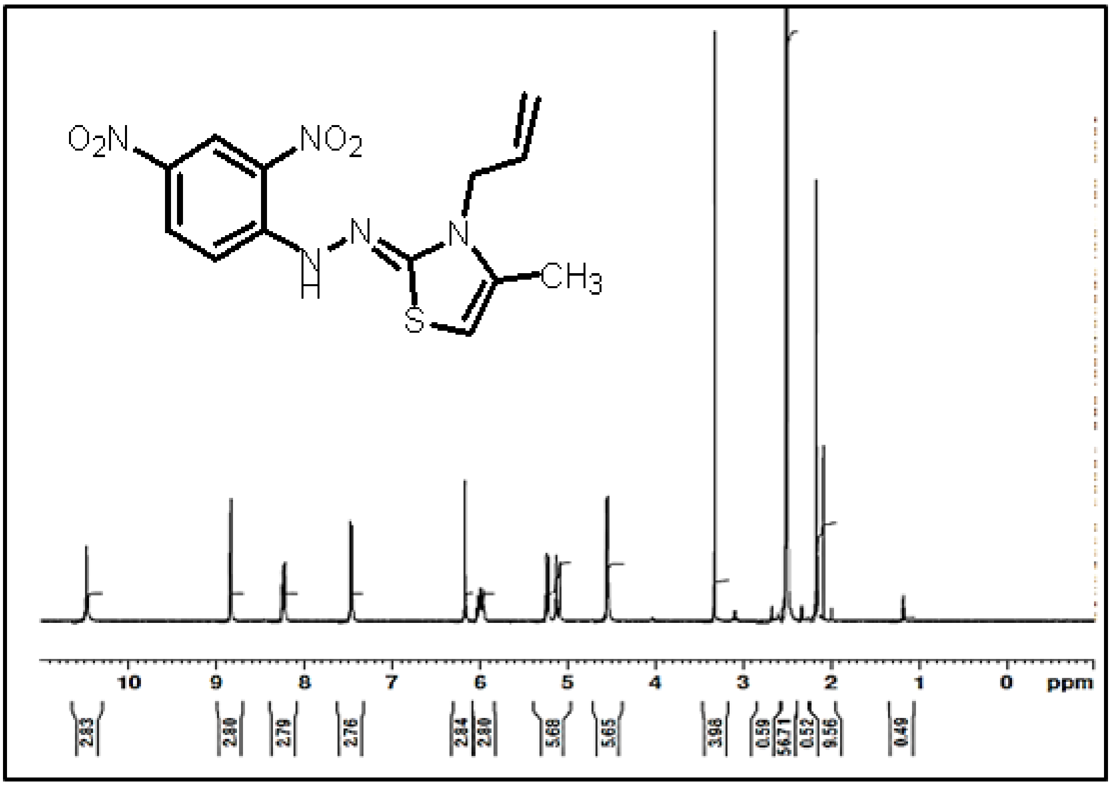

Figure S13: ^{13}H NMR spectrum of **3c**

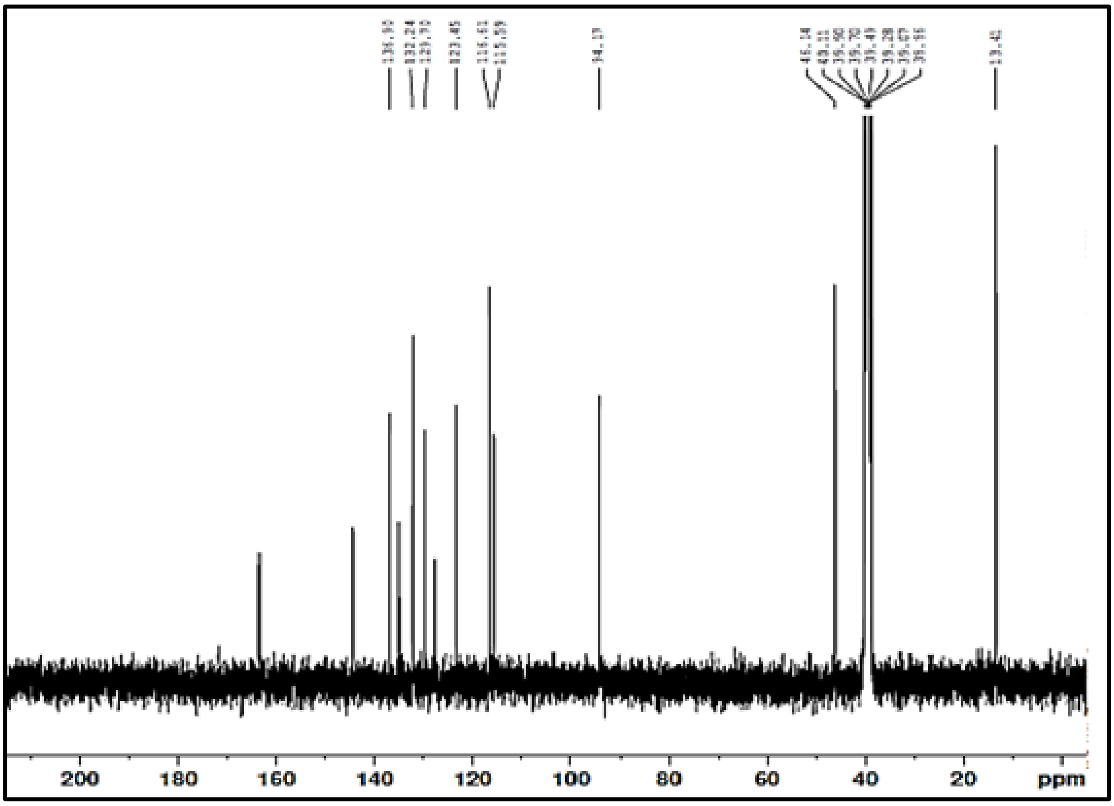

Spectral data of compound **3d**:

Figure S14: IR spectrum of **3d**

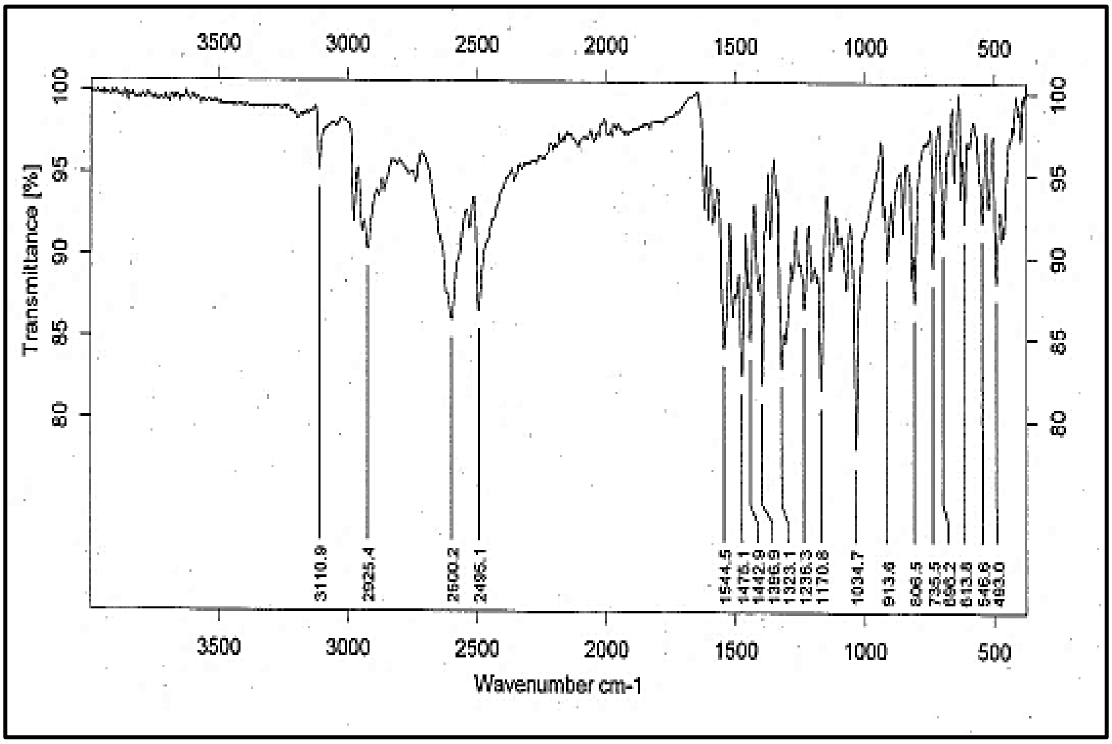

Figure S15: Mass spectrum of **3d**

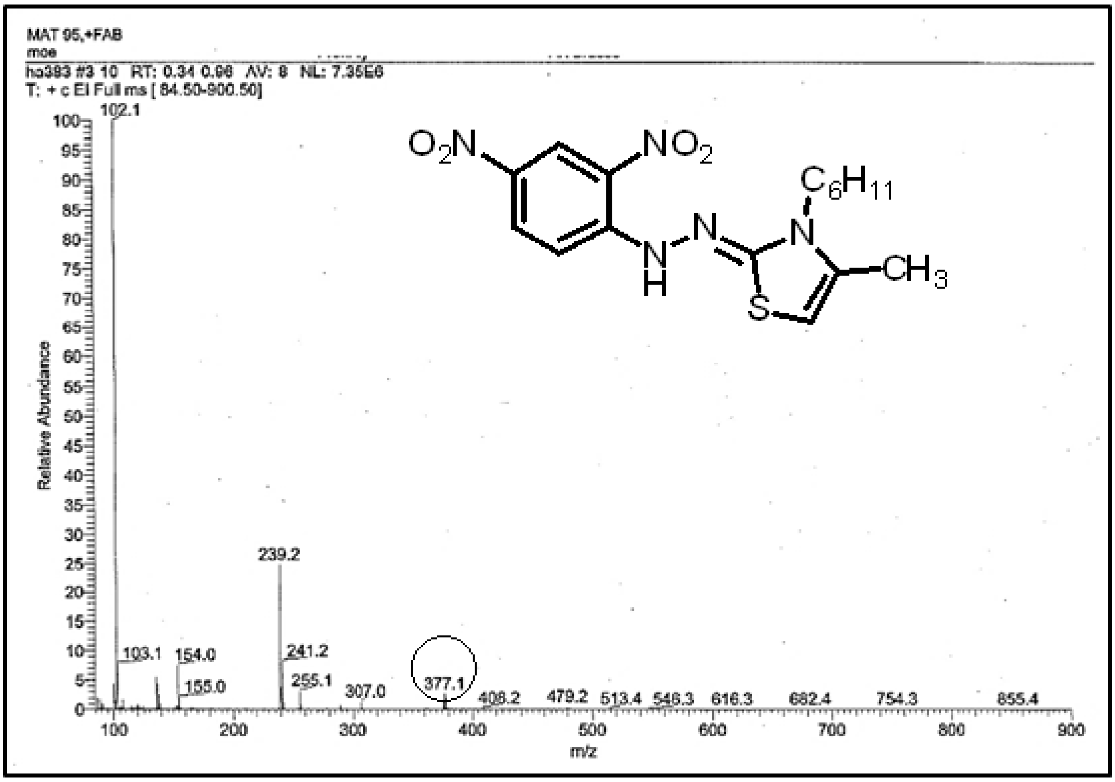

Figure S16: ^{1}H NMR of **3d**

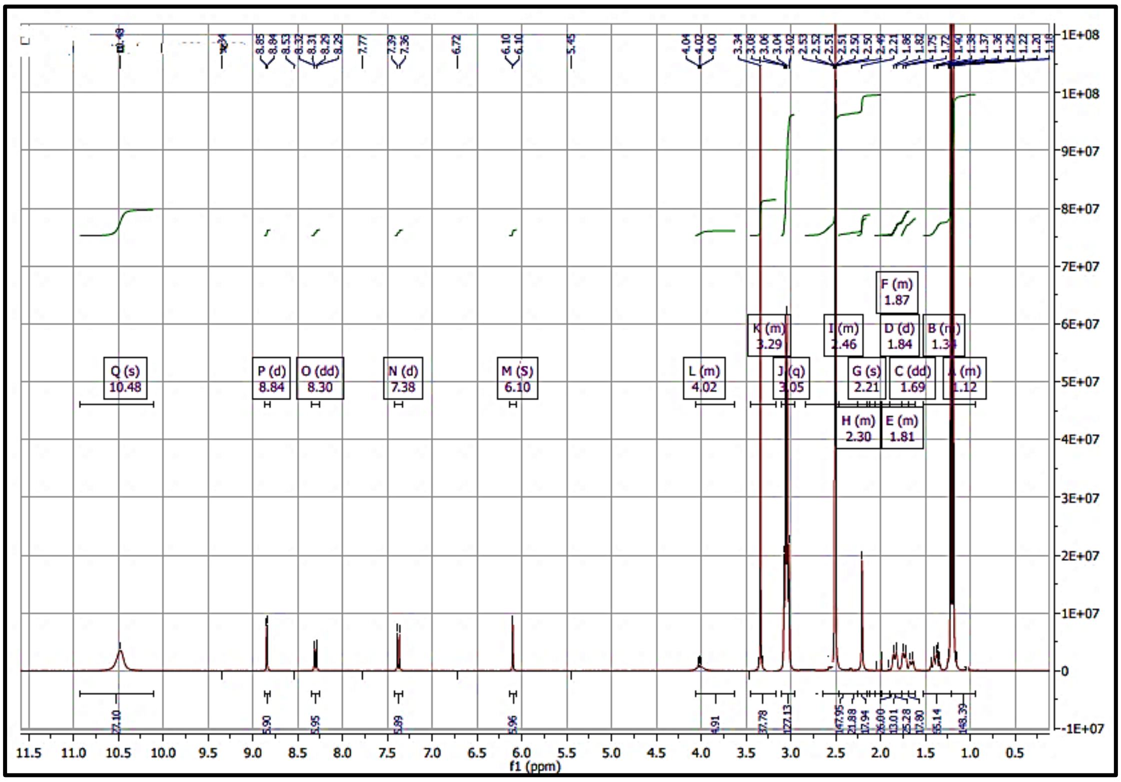

Spectral data of compound **3e**:

Figure S17: IR spectrum of **3e**

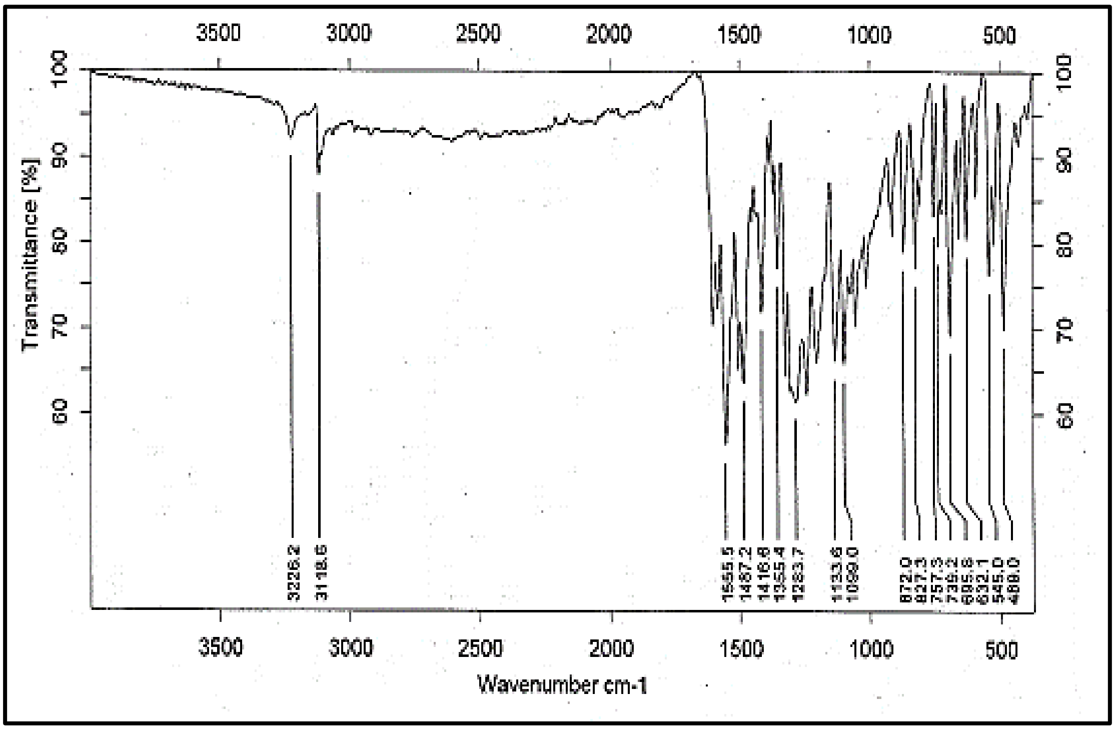

Figure S18: Mass spectrum of **3e**

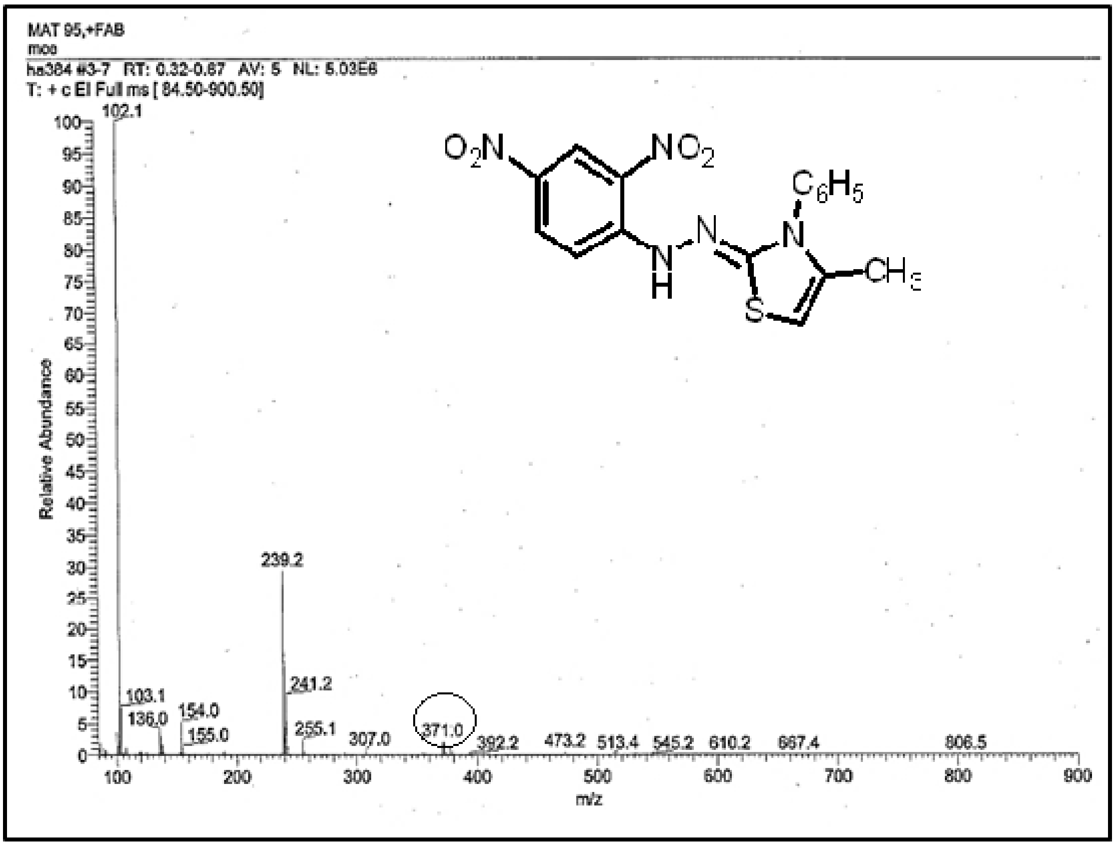

Figure S19: ^{1}H NMR of **3e**

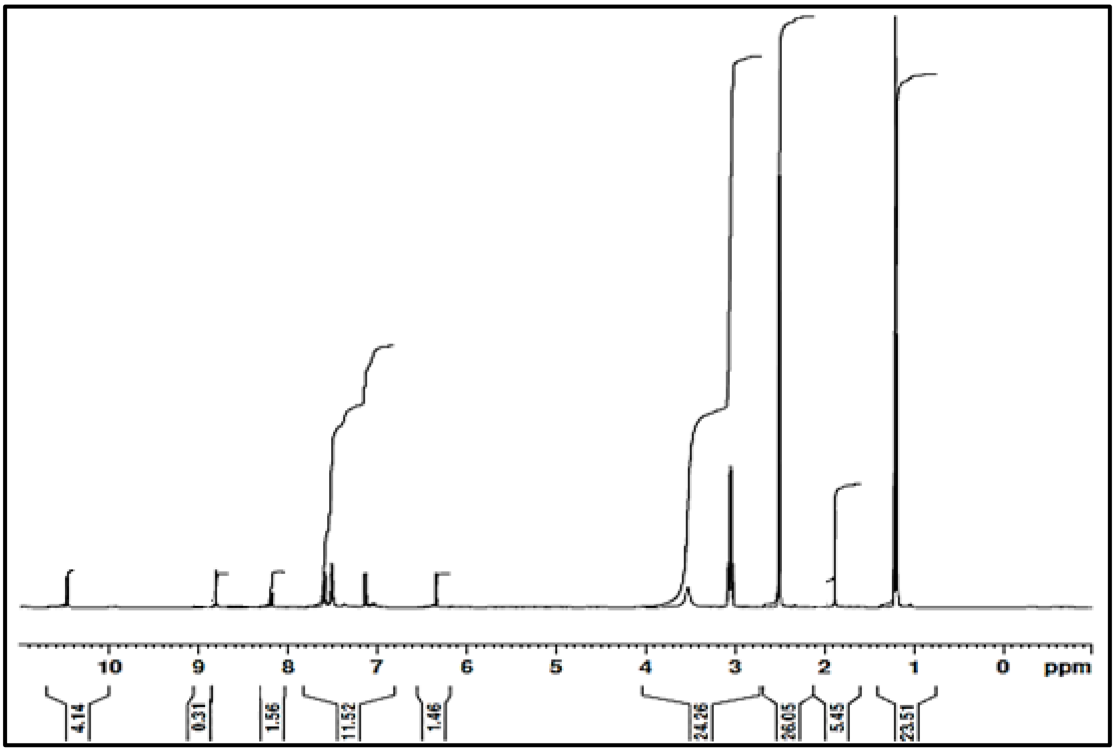

Figure S20: ^{13}C NMR of **3e**

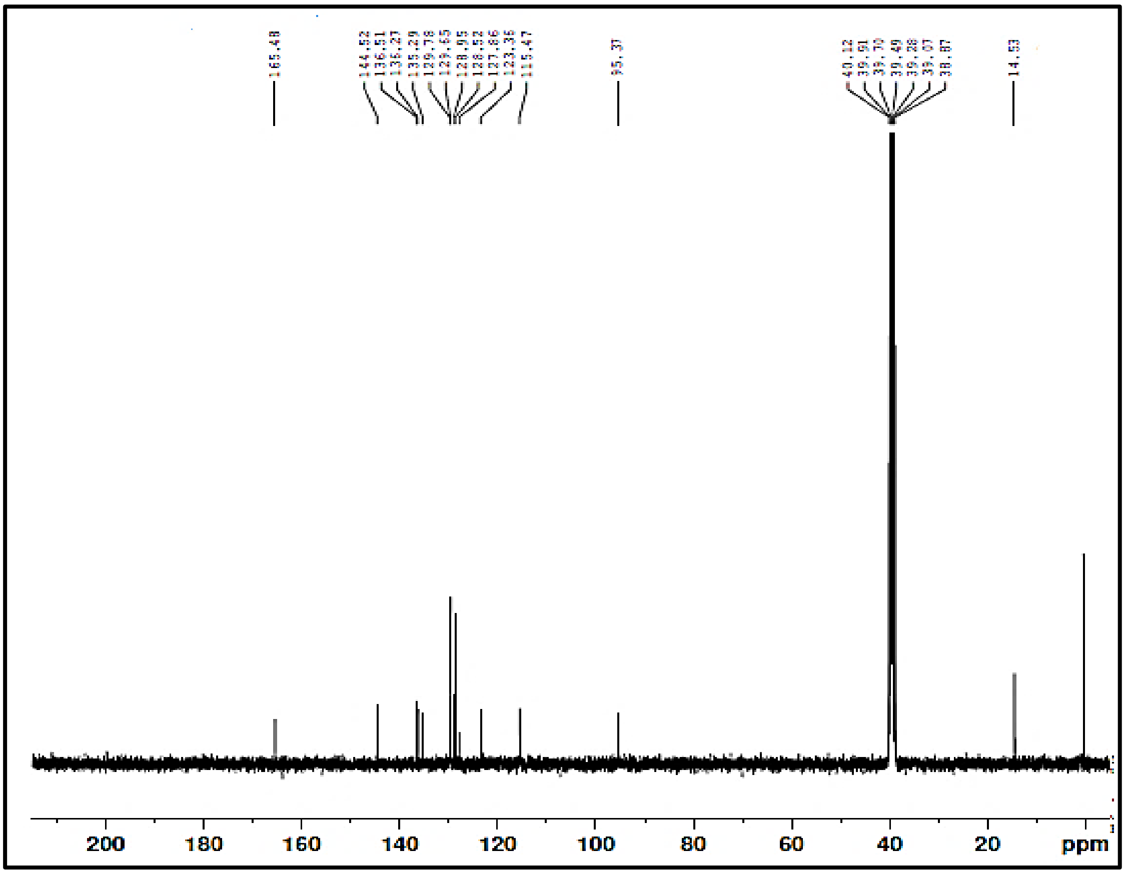

Spectral data of compound **3f**:

Figure S21: IR spectrum of **3f**

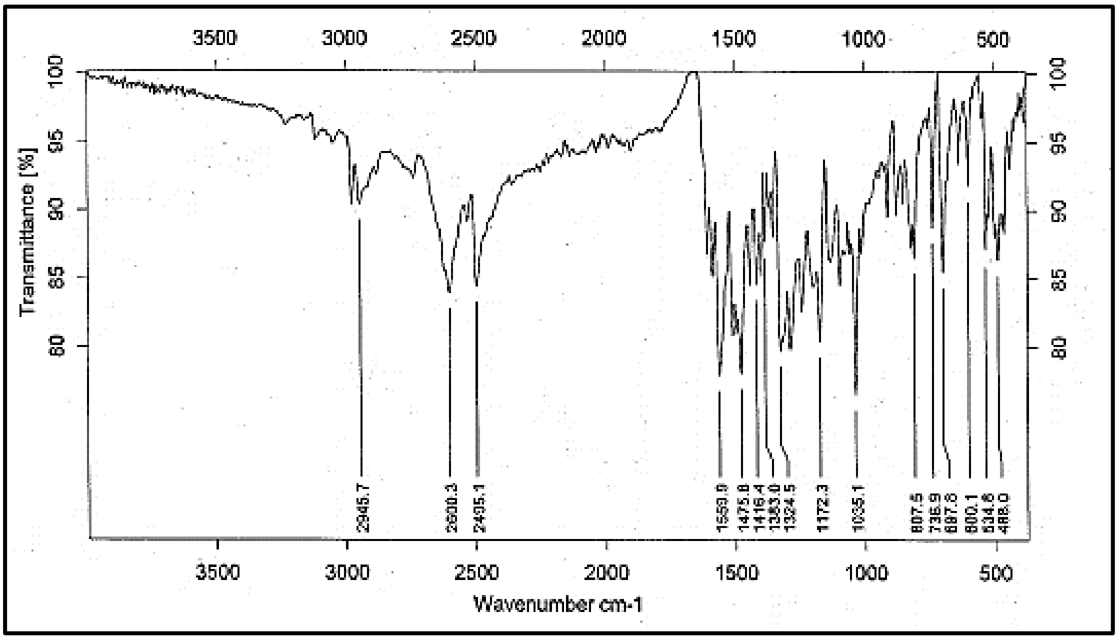

Figure S22: Mass spectrum of **3f**

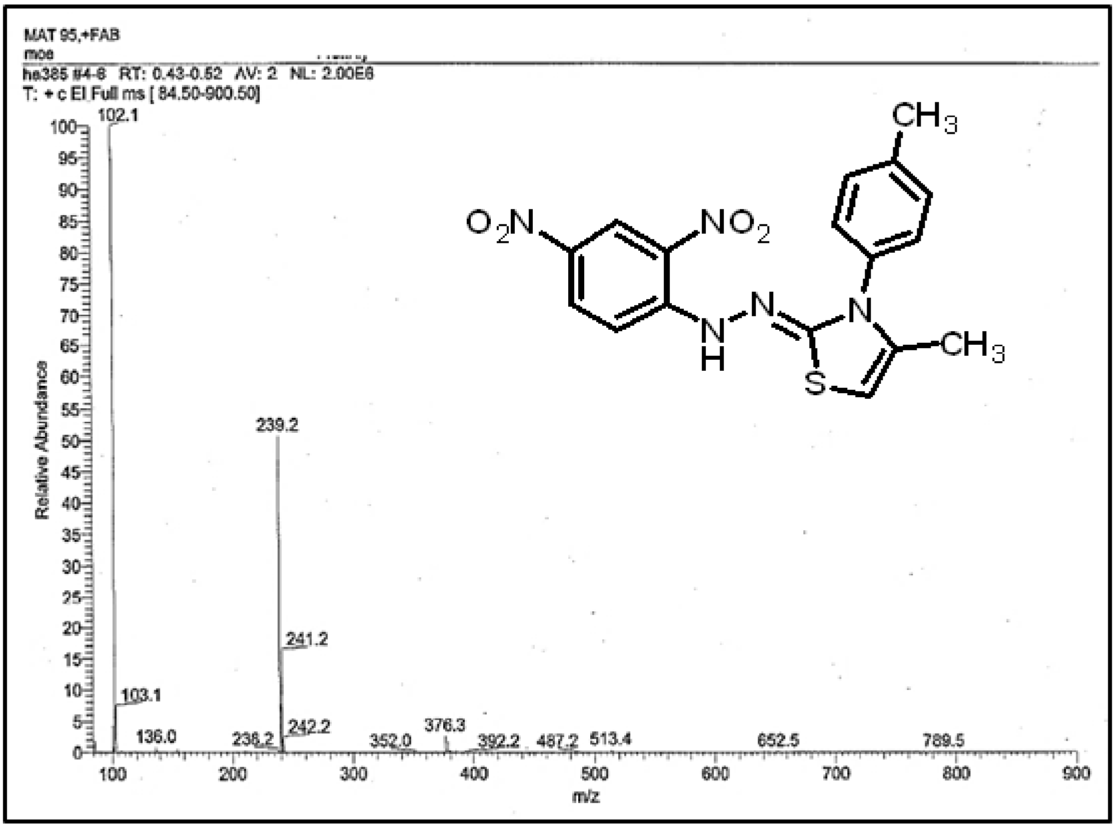

Figure S23: ^{1}H NMR of **3f**

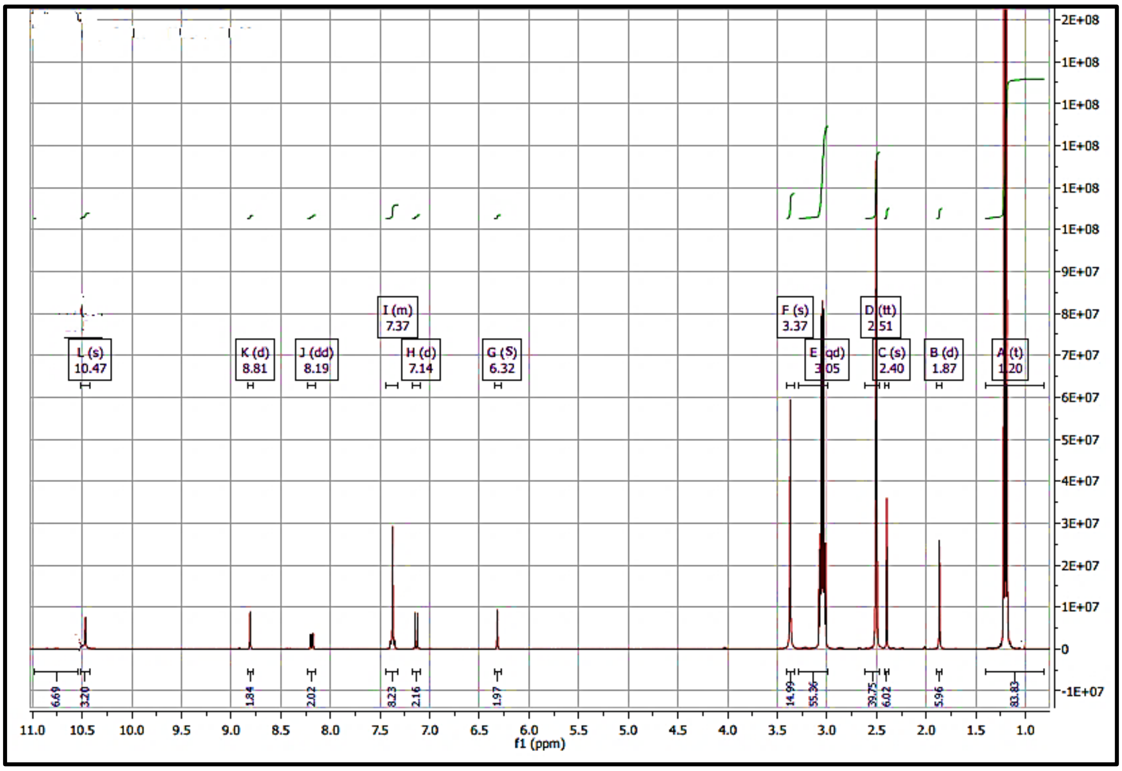

Figure S24: ^{13}C NMR of **3f**

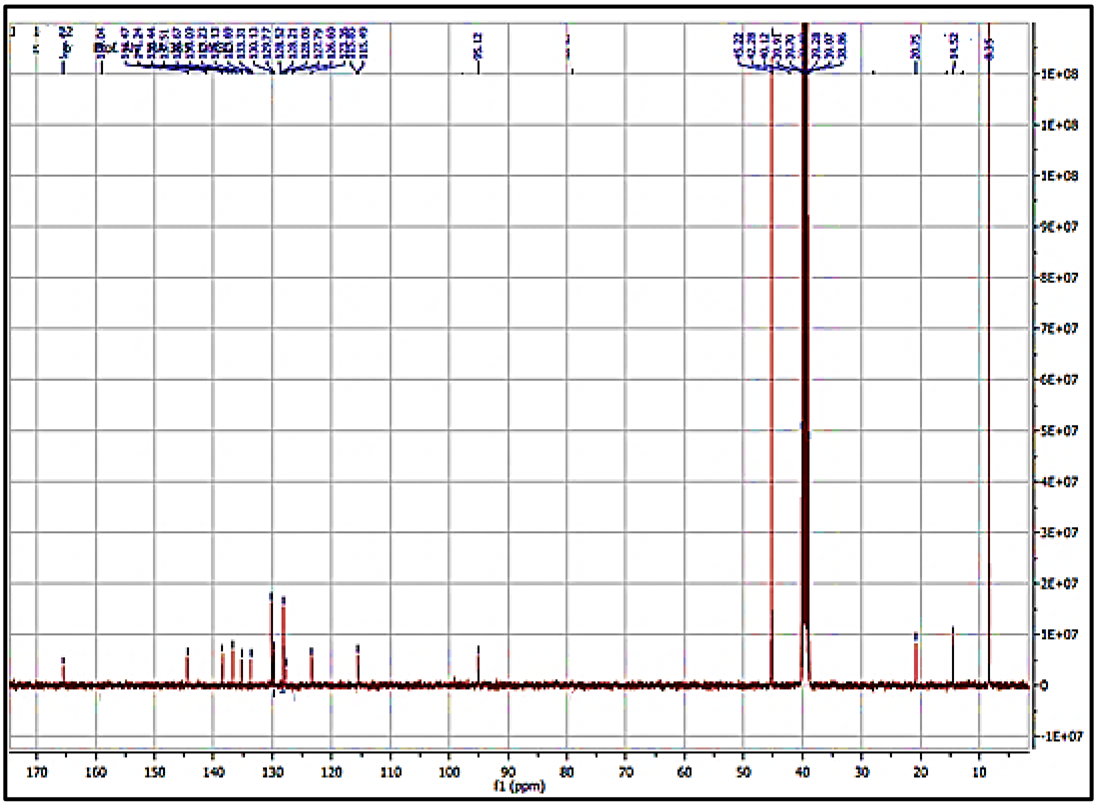

Spectral data of compound **3g**:

Figure S25: IR spectrum of **3g**

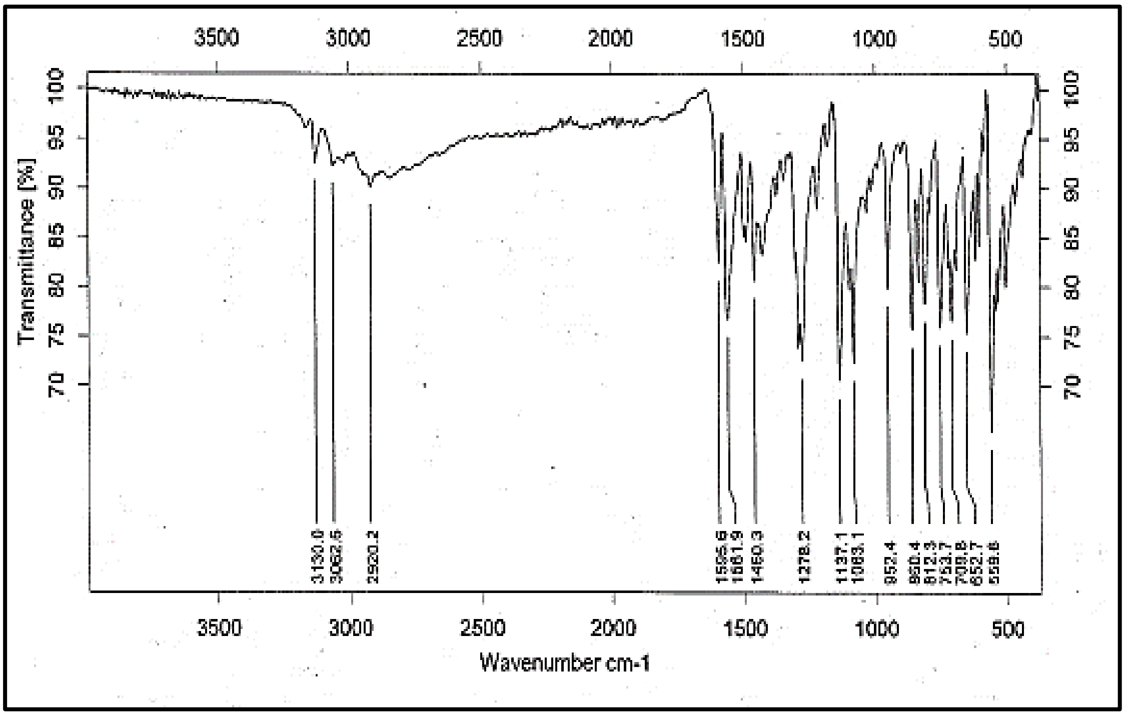

Figure S26: Mass spectrum of **3g**

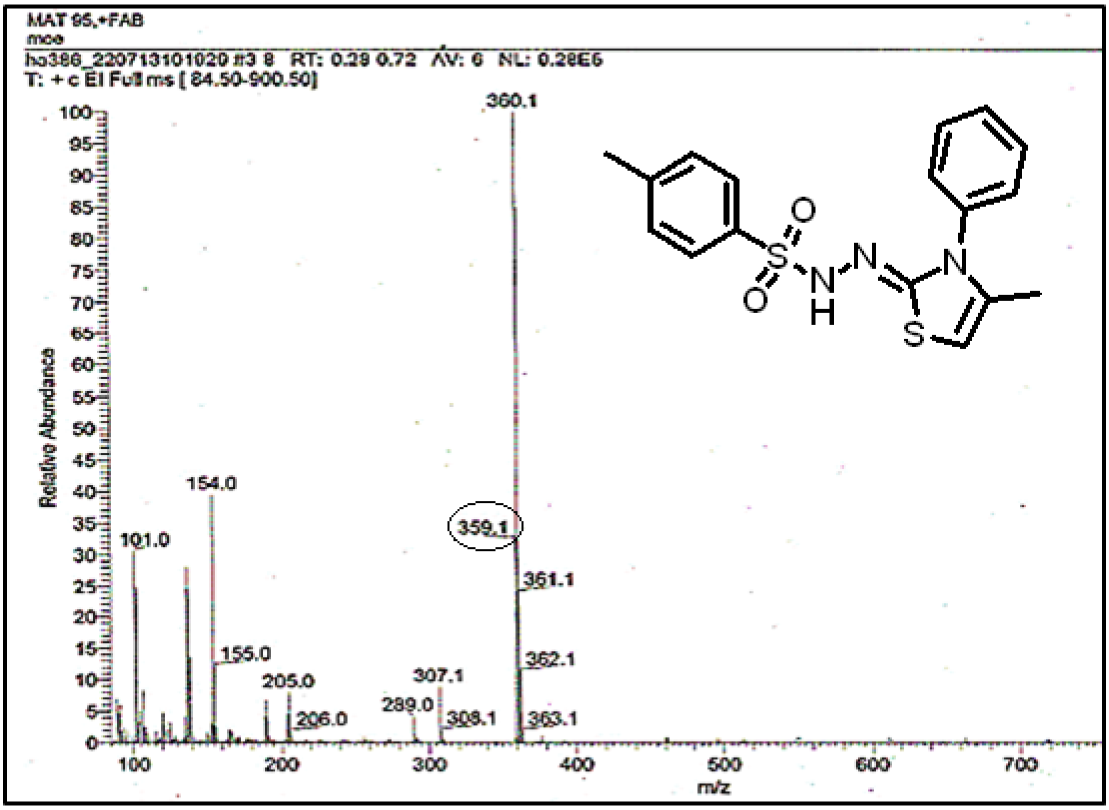

Figure S27: ^{1}H NMR of **3g**

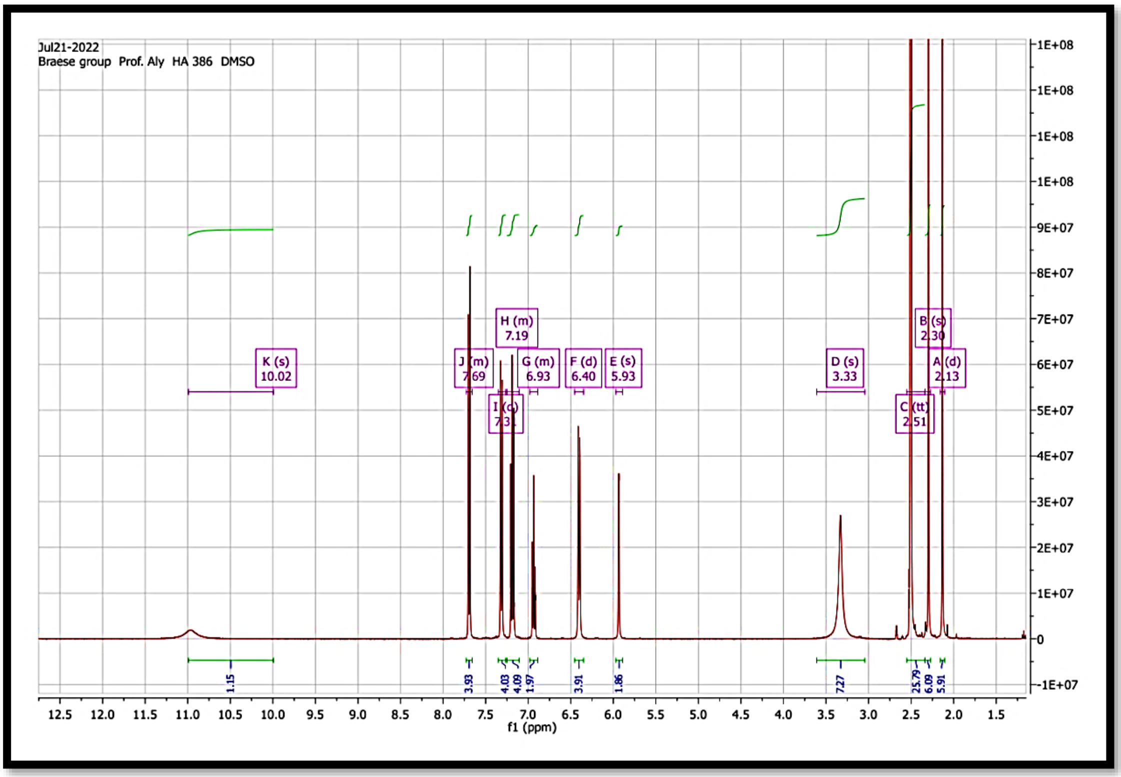

Figure S28: ^{13}C NMR of **3g**

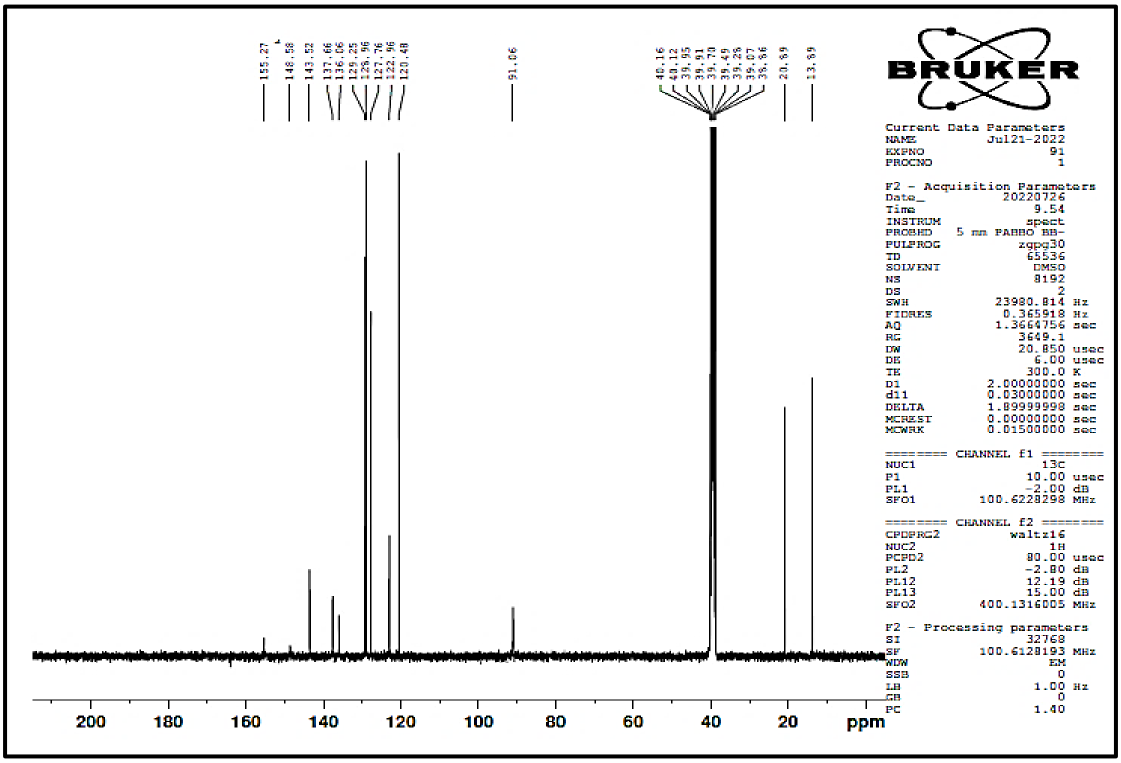

Spectral data of compound **3h**:

Figure S29: IR spectrum of **3h**

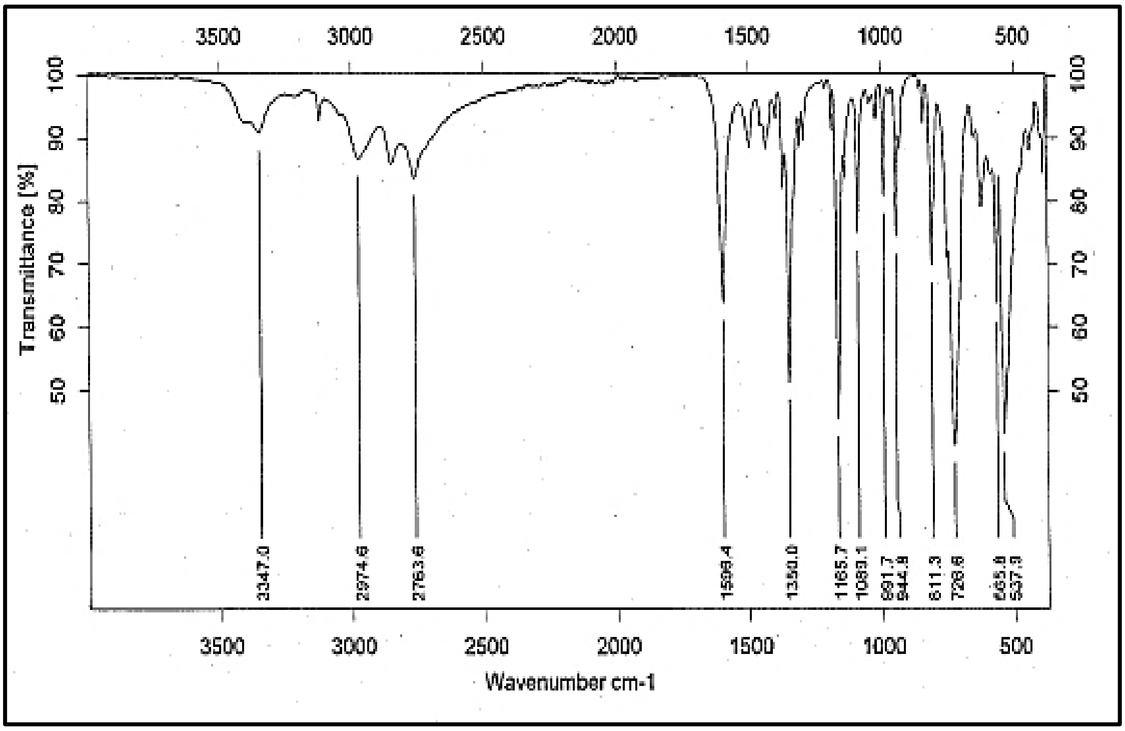

Figure S30: Mass spectrum of **3h**

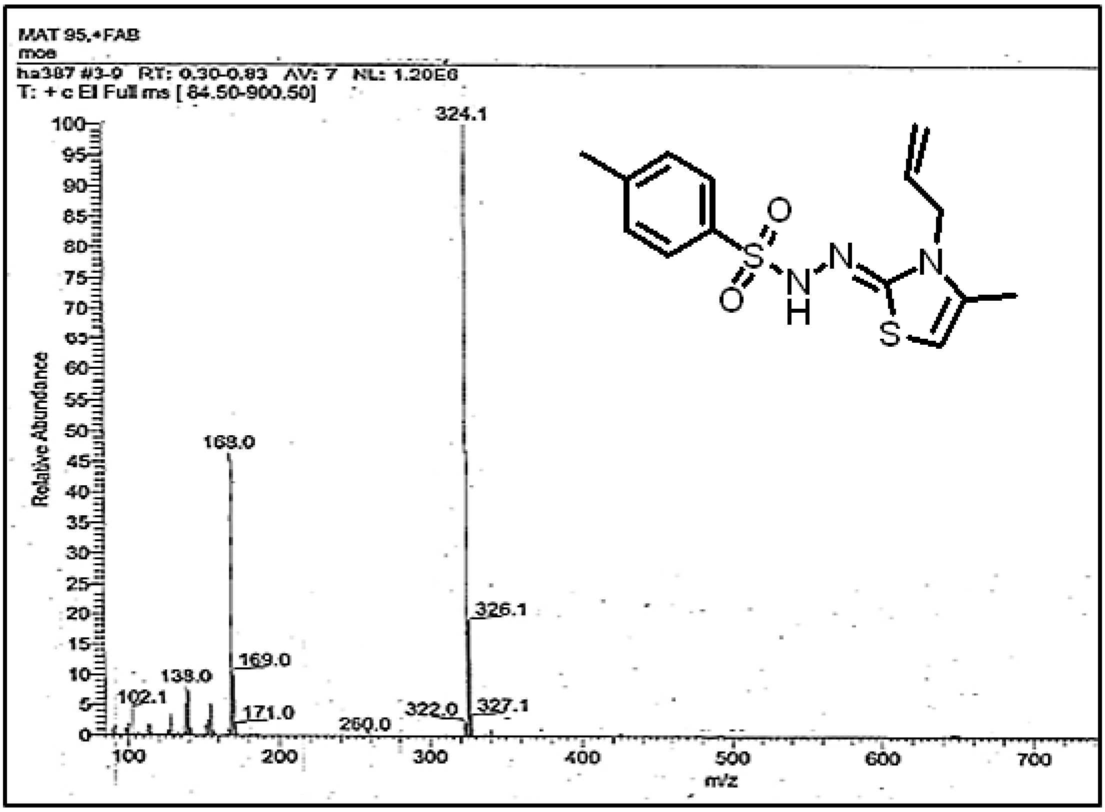

Spectral data of compound **3i**:

Figure S31: IR spectrum of **3i**

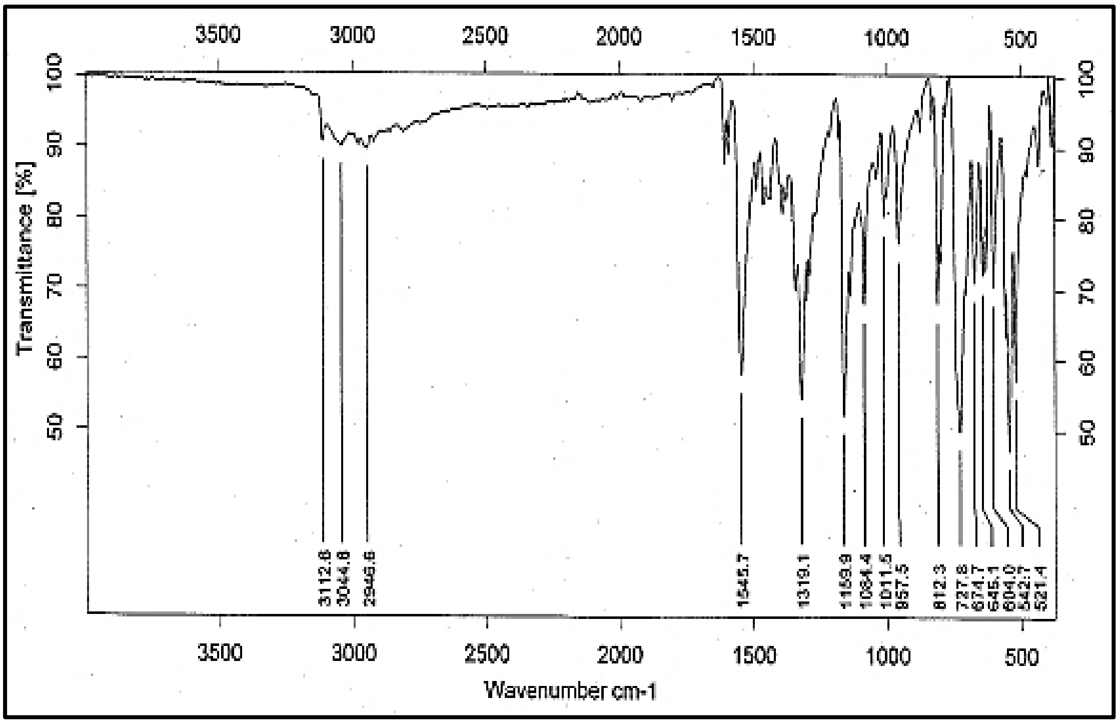

Figure S32: Mass spectrum of **3i**

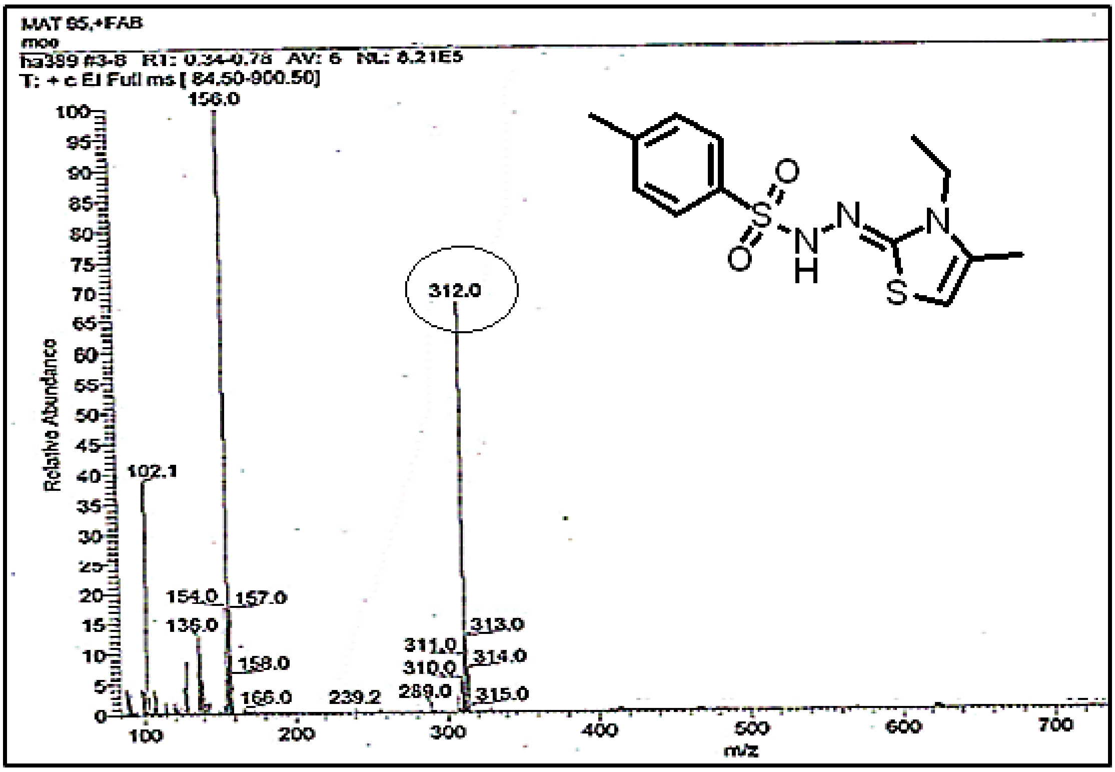

4. EXPERIMENTAL

4.1. Chemistry

General Details

Melting points were determined using open glass capillaries on an SMP30 melting point apparatus and uncorrected. IR spectra were recorded from potassium bromide disks on Alpha, Bruker FT-IR. ^{1}H and ^{13}C NMR spectra (400 MHz for ^{1}H, 100 MHz for ^{13}C) were observed in DMSO-d_6 as a solvent on Varian mercury plus 300 spectrometers with tetramethyl silane as an internal standard. Mass spectra were obtained in Varian MAT 95, FAB+ doubly focusing instrument using electron impact ionization (70 eV). TLC was performed as analytical Merck 9385 silica aluminum sheets (Kieselgel 60) with pf$_{254}$ indicator; TLCs were viewed at λ_{max} = 254 nm. Elemental analyses for C, H, N, and S were carried out using an Elmer 306.

4.2. Biological evaluation

4.2.1. Cytotoxic activity using MTT Assay and evaluation of IC_{50}

4.2.1.1. MTT assay

MTT assay was performed to investigate the effect of the synthesized compounds on mammary epithelial cells (MCF-10A). The cells were propagated in medium consisting of Ham's F-12 medium/ Dulbecco's modified Eagle's medium (DMEM) (1:1) supplemented with 10% foetal calf serum, 2 mM glutamine, insulin (10 μg/mL), hydrocortisone (500 ng/mL) and epidermal growth factor (20 ng/mL). Trypsin ethylenediamine tetra acetic acid (EDTA) was used to passage the cells after every 2-3 days. 96-well flat-bottomed cell culture plates were used to seed the cells at a density of 104 cells mL^{-1}. The medium was aspirated from all the wells of culture plates after 24 h followed by the addition of synthesized compounds (in 200 μL medium to yield a final concentration of 0.1% (v/v) dimethyl sulfoxide) into individual wells of the plates. Four wells were designated to a single compound. The plates were allowed to incubate at 37°C for 96 h. Afterwards, the medium was aspirated and 3-[4,5-dimethylthiazol-2-yl]-2,5-diphenyltetrazolium bromide (MTT) (0.4 mg/mL) in medium was added to each well and subsequently incubated for 3 h. The medium was aspirated and 150 μL dimethyl sulfoxide (DMSO) was added to each well. The plates were vortexed followed by the measurement of absorbance at 540 nm on a microplate reader. The results were presented as inhibition (%) of proliferation in contrast to controls comprising 0.1% DMSO.

4.2.1.2. Assay for antiproliferative effect

To explore the antiproliferative potential of compounds propidium iodide fluorescence assay was performed using different cell lines such as Panc-1 (pancreas cancer cell line), MCF-7 (breast cancer cell line), HT-29 (colon cancer cell line) and A-549 (epithelial cancer cell line), respectively. To calculate the total nuclear DNA, a fluorescent dye (propidium iodide, PI) is used which can attach to the DNA, thus offering a quick and precise technique. PI cannot pass through the cell membrane and its signal intensity can be considered as directly proportional to quantity of cellular DNA. Cells whose cell membranes are damaged or have changed permeability are counted as dead ones. The assay was performed by seeding the cells of different cell lines at a density of 3000-7500 cells/well (in200μl medium) in culture plates followed by incubation for 24h at 37 °C in humidified 5%CO2/95% air atmospheric conditions. The medium was removed; the compounds were added to the plates at 10 μM concentrations (in 0.1% DMSO) in triplicates, followed by incubation for 48h. DMSO (0.1%) was used as control. After incubation, medium was removed followed by the addition of PI (25 μl, 50μg/mL in water/medium) to each well of the plates. At -80 °C, the plates were allowed to freeze for 24 h, followed by thawing at 25oC. A fluorometer (Polar-Star BMG Tech) was used to record the readings at excitation and emission wavelengths of 530 and 620 nm for each well. The percentage cytotoxicity of compounds was calculated using the following formula:

$$\%\ \text{Cytotoxicity} = \frac{A_C - A_{TC}}{A_C} \times 100$$

Where A*TC*= Absorbance of treated cells and AC= Absorbance of control. Erlotinib was used as positive control in the assay.

4.2.2. EGFR inhibitory assay

Baculoviral expression vectors including pBlueBacHis2B and pFASTBacHTc were used separately to clone 1.6 kb cDNA coding for EGFR cytoplasmic domain (EGFR-CD, amino acids 645–1186). 5′ upstream to the EGFR sequence comprised a sequence that encoded (His)6. Sf-9 cells were infected for 72h for protein expression. The pellets of Sf-9 cells were solubilized in a buffer containing sodium vanadate (100 μM), aprotinin (10 μg/mL), triton (1%), HEPES buffer(50mM), ammonium molybdate (10 μM), benzamidine HCl (16 μg/mL), NaCl (10 mM),leupeptin (10 μg/mL) and pepstatin (10 μg/mL) at 0°C for 20 min at pH 7.4, followed by centrifugation for 20 min. To eliminate the nonspecifically bound material, a Ni-NTA super flow packed column was used to pass through and wash the crude extract supernatant first with 10mM and then with 100 mM imidazole. Histidine-linked proteins were first eluted with 250 and then with 500 mM imidazole subsequent to dialysis against NaCl (50 mM), HEPES (20 mM), glycerol (10%) and 1 μg/mL each of aprotinin, leupeptin and pepstatin for 120 min. The purification was performed either at 4 °C or on ice. To record autophosphorylation level, EGFR kinase assay was carried out on the basis of DELFIA/Time-Resolved Fluorometry. The compounds were first dissolved in DMSO absolute, subsequent to dilution to appropriate concentration using HEPES (25 mM) at pH 7.4. Each compound (10 μL) was incubated with recombinant enzyme (10 μL, 5 ng for EGFR, 1:80 dilution in 100 mM HEPES) for 10 min at 25°C, subsequent to the addition of 5X buffer (10 μL, containing 2 mM MnCl2, 100 μM Na3VO4, 20 mM HEPES and 1 mM DTT) and ATP-MgCl2 (20 μL, containing 0.1 mM ATP and 50 mM MgCl2) and incubation for 1h. The negative and positive controls were included in each plate by the incubation of enzyme either with or without ATP-MgCl2. The liquid was removed after incubation and the plates were washed thrice using wash buffer.

Europium-tagged antiphosphotyrosine antibody (75 µL, 400 ng) was added to each well followed by incubation of 1h and then washing of the plates using buffer. The enhancement solution was added to each well and the signal was recorded at excitation and emission wavelengths of 340 at 615 nm. The autophosphorylation percentage inhibition by compounds was calculated using the following equation:

$$100\% - [(negative\ control)/(positive\ control) - (negative\ control)]$$

Using the curves of percentage inhibition of eight concentrations of each compound, IC50 was calculated. Majority of signals detected by antiphosphotyrosine antibody were from EGFR because the enzyme preparation contained low impurities.

4.2.3. BRAF^{V600E} inhibitory assay

V^{600E} mutant BRAF kinase assay was performed to investigate the activity of tested compounds against BRAF. Mouse full-length GST-tagged BRAF^{V600E} (7.5 ng, Invitrogen, PV3849) was pre-incubated with drug (1 µL) and assay dilution buffer (4 µL) for 60 min at 25°C. In assay dilution buffer, a solution (5 µL) containing $MgCl_2$ (30 mM), ATP (200 µM), recombinant human full length (200 ng) and *N*-terminal His-tagged MEK1 (Invitrogen) was added to start the assay, subsequent to incubation for 25 min at 25°C. The assay was stopped using 5X protein denaturing buffer (LDS) solution (5 µL). To further denature the protein, heat (70° C) was applied for 5 min. 4-12% precast NuPage gel plates (Invitrogen) were used to carry out electrophoresis (at 200 V). 10 µL of each reaction was loaded into the precast plates and electrophoresis was allowed to proceed. After completion of electrophoresis, the front part of the precast gel plate (holding hot ATP) was cut and afterwards cast-off. The dried gel was developed using a phosphor screen. A reaction without active enzyme was used as negative control while that containing no inhibitor served as positive control. To study

the effect of compounds on cell-based pERK1/2 activity in cancer cells, commercially available ELISA kits (Invitrogen) were used according to manufacturer's instructions.

4.3. Statistical analysis

Computerized Prism 5 program was used to statistically analyzed data using one-way ANOVA test followed by Tukey's as post ANOVA for multiple comparison at $P \leq .05$. Data were presented as mean ± SEM.
